# Deep Sequencing Reveals Differences in the Transcriptional Landscapes of Fibers from Two Cultivated Species of Cotton

**DOI:** 10.1371/journal.pone.0048855

**Published:** 2012-11-15

**Authors:** Jean-Marc Lacape, Michel Claverie, Ramon O. Vidal, Marcelo F. Carazzolle, Gonçalo A. Guimarães Pereira, Manuel Ruiz, Martial Pré, Danny Llewellyn, Yves Al-Ghazi, John Jacobs, Alexis Dereeper, Stéphanie Huguet, Marc Giband, Claire Lanaud

**Affiliations:** 1 CIRAD, UMR AGAP, Montpellier, France; 2 University of CampinasLaboratory of Genomics and Expression, Campinas, SP, Brazil; 3 CSIRO Plant Industry, Canberra, ACT, Australia; 4 Bayer BioScience N.V., Ghent, Belgium; 5 IRD, UMR RPB, Montpellier, France; 6 URGV, UMR INRA 1165, Evry, France; 7 EMBRAPA Algodão, Núcleo Cerrado da Embrapa Algodão, Santo Antônio de Goiás, GO, Brazil; Kansas State University, United States of America

## Abstract

Cotton (*Gossypium*) fiber is the most prevalent natural product used in the textile industry. The two major cultivated species, *G. hirsutum* (*Gh*) and *G. barbadense* (*Gb*), are allotetraploids with contrasting fiber quality properties. To better understand the molecular basis for their fiber differences, EST pyrosequencing was used to document the fiber transcriptomes at two key development stages, 10 days post anthesis (dpa), representing the peak of fiber elongation, and 22 dpa, representing the transition to secondary cell wall synthesis. The 617,000 high quality reads (89% of the total 692,000 reads) from 4 libraries were assembled into 46,072 unigenes, comprising 38,297 contigs and 7,775 singletons. Functional annotation of the unigenes together with comparative digital gene expression (DGE) revealed a diverse set of functions and processes that were partly linked to specific fiber stages. Globally, 2,770 contigs (7%) showed differential expression (>2-fold) between 10 and 22 dpa (irrespective of genotype), with 70% more highly expressed at 10 dpa, while 2,248 (6%) were differentially expressed between the genotypes (irrespective of stage). The most significant genes with differential DGE at 10 dpa included expansins and lipid transfer proteins (higher in *Gb*), while at 22 dpa tubulins, cellulose, and sucrose synthases showed higher expression in *Gb*. DGE was compared with expression data of 10 dpa-old fibers from Affymetrix microarrays. Among 543 contigs showing differential expression on both platforms, 74% were consistent in being either over-expressed in *Gh* (242 genes) or in *Gb* (161 genes). Furthermore, the unigene set served to identify 339 new SSRs and close to 21,000 inter-genotypic SNPs. Subsets of 88 SSRs and 48 SNPs were validated through mapping and added 65 new loci to a RIL genetic map. The new set of fiber ESTs and the gene-based markers complement existing available resources useful in basic and applied research for crop improvement in cotton.

## Introduction

The two major cultivated cotton species, *Gossypium hirsutum* (over 90% of world’s production) and *G. barbadense* (7%), are allotetraploids with an AD (2*n* = 52) genome constitution. They originated from an hybridization between an A genome (2*n* = 26) species much like modern *G. arboreum* and *G. herbaceum*, and a D genome (2*n* = 26) species similar to modern *G. raimondii* [1]. *G. hirsutum* comprises cultivars of high yield potential and broad adaptability, that produce fibers of acceptable processing quality suited to general purpose textiles, while *G. barbadense* comprises cultivars of medium to low yield but producing fibers of excellent quality, being extremely long, fine and strong and suited to the premium textile market. Cotton fibers are trichome-like single cells derived from the epidermis of the outer seed coat [2]. Fiber morphogenesis can be divided into four distinct, but overlapping stages: initiation, elongation, secondary cell wall (SCW) synthesis, and maturation (desiccation). During fiber elongation (3–20 days post anthesis, dpa), the most rapid growth occurs around 10–12 dpa, while the transition from primary to secondary wall deposition starts around 16–20 dpa, with cellulose synthesis as the major cellular process thereafter [3]. Cotton fibers can elongate to 3–5 cm depending on the species, rendering them one of the longest and fastest growing cell types in the plant kingdom [2]. Mature and dry cotton fibers contain about 90% cellulose, most of which comprises the secondary cell wall. Cotton fiber has attracted the most attention from functional genomics, as highlighted by the plethora of cotton genes isolated from ovules at the pre-flowering stage through to maturing fibers [4]–[6]. The development of Expressed Sequence Tag (EST) collections and microarray platforms have also been used to explore predominantly fiber expressed genes [7]–[9] and various gene functional categories have been assigned to some of the different fiber development stages [10]. In terms of physiological and cellular processes, cotton fiber elongation is the result of a complex interplay between cell turgor and cell wall extensibility, requiring the involvement of various transport, catabolic, biosynthetic and signaling pathways [11]. High transcription factor activity and expression of phytohormonal regulators are associated with the early stages of fiber development [8], [12]. Cellulose synthesis is the predominant event in fiber cells in the SCW synthesis stage, but this SCW stage has received relatively little attention at the genome level because of the difficulties in working with the highly vacuolated fiber cells at this stage [13]. Most of the genomics research on cotton fiber has also been undertaken on *G. hirsutum* and its different mutant types, such as the fiberless/lintless and short fiber mutants (e.g., [12], [14], [15]). Relatively few transcriptome studies have investigated the cellular mechanisms and genes underlying the important fiber developmental and phenotypic differences between the two major cultivated species *G. hirsutum* and *G. barbadense* [16]–[18], despite these being important in many cotton improvement programs. It has been reported previously [18] that fiber development proceeds with relatively similar timelines in representative accessions of *Gh* and *Gb* under glasshouse conditions.

ESTs represent a valuable sequence resource for comprehensive transcriptome analyses, genome annotation, accelerating gene discovery, large-scale expression analyses, and for facilitating breeding objectives by providing markers tagging specific genes, such as EST-SSRs and SNPs. Currently, there are over 5 million ESTs (including Sanger and 454 sequences, but excluding the rapidly increasing amounts of Illumina short read data) of *Gossypium* spp. in Genbank. Among the published EST libraries, the majority are from ovules or developing fibers. Species representation includes both tetraploid and diploid cotton, although *G. barbadense* is well under-represented. Several significant cotton EST assemblies have been released, including those by the Gene Index Project (Cotton Gene Index Release 11.0 from http://compbio.dfci.harvard.edu/tgi/plant.html), with 50,873 Tentative Consensus contigs and 67,119 singletons assembled from over 354,000 Sanger ESTs, and by the project Comparative Evolutionary Genomics of Cotton (http://cottonevolution.info/), including the most recent hybrid assembly (Sanger and 454-derived sequences), released under the acronym Cotton46, which contains approximately 4.4 million Sanger and 454 EST reads and comprises 44,900 contigs assembled from multiple *Gossypium* species. At the time of this manuscript no completed assembly of the tetraploid cotton genome sequence has been published, although several sequencing projects are well underway [19] and two sequence assemblies of the diploid D genome, *G. raimondii* have recently been made public ([20] and http://www.phytozome.net/cotton.php).

Transcript abundance information can be captured using a variety of techniques ranging from RT-PCR through cDNA microarray hybridisation to next-generation sequencing (NGS, RNA-Seq) technologies. The increasing throughput of NGS technologies, in particular, shows great potential for cost–effective large-scale generation of ESTs and has already been used in several plant species [21], [22]. High-throughput transcriptome sequencing has not only accelerated research in comparative genomics and biodiversity studies, but can also be used to quantify gene expression by counting individual sequence reads unambiguously mapped to the corresponding transcript [23] and is rapidly replacing microarrays as the technique of choice for global gene expression analysis. Recent examples of *in-silico* expression analysis (also called digital gene expression, DGE, analysis) between genotypes and across different biological states or treatments in plants using NGS transcripts sequencing include *Siraitia* [24], based on Illumina/Solexa sequencing, chestnut [25], olive [26], eucalyptus [27], watermelon [28] and cucumber [29], all based on 454 sequencing. Some challenges still remain, particularly in polyploid species like cotton with multiple closely related genomes, but enhanced bioinformatic tools are being developed to expedite some of the difficulties in assembling short reads to the appropriate homoeologs [30], [31].

We recently reported the development of an inter-specific *G. hirsutum* x *G. barbadense* RIL population [32] which was used for QTL mapping of fiber quality traits [33] as well as for the mapping of expression QTLs (eQTLs) using cDNA-AFLP [34] and microarray hybridization (manuscript in preparation) to identify genes underlying fiber quality. In this study we further characterize the fiber transcriptomes of the two parental genotypes of that population. We used 454 pyrosequencing to characterize cDNAs from developing fibers at two key developmental time-points. A unigene set was assembled and annotated, and differential DGE was assessed from the different time-point and genotype representations of the reads within assembled contigs. As a complementary approach, we conducted microarray-based hybridization profiling using the cotton Affymetrix gene chip and labeled cDNAs from the same two genotypes and compared differentially expressed genes identified by the two platforms. The 454 unigenes were also mined for the presence of microsatellite repeats and SNPs.

## Results

### EST Sequencing and Assembly of an Expanded Gossypium Fiber Transcriptome

In this study we examined the transcriptomes of developing fibers of two cotton accessions representing the most important fiber producing *Gossypium* species, *G. hirsutum* and *G. barbadense*, focusing on two key developmental stages, peak fiber elongation and the onset of secondary cell wall biogenesis, i.e. 10 and 22 dpa respectively. Detailed statistics for the four 454 EST libraries, combining 2 species *Gh* and *Gb* and 2 fiber development stages, 10 and 22 dpa (referred to throughout as Gh10, Gb10, Gh22 and Gb22) are given in Table 1. One of the 4 libraries, Gb22, generated a lower number of reads of slightly higher mean length (Table 1).

**Table 1:** Statistics of sequencing and assembly data.

Pyrosequencing Library	Gh10	Gb10	Gh22	Gb22	Total
Genotype	GUA	VH8	GUA	VH8	
Species	*G. hirsutum*	*G. barbadense*	*G. hirsutum*	*G. barbadense*	
Stage	10 dpa	10 dpa	22 dpa	22 dpa	
No. reads (trimmed)	175,657	197,383	194,251	124,580	691,871
No. bases (Mbp)	60,6	66,8	70,1	46,5	244
Mean length (bp)	345	338	361	373	353
No. reads <100 bp	22,292	19,298	20,899	12,084	74,573
%	13%	10%	11%	10%	
High quality reads	153,365	178,085	173,352	112,496	617,298
Assembly					
No. contigs	25,773	25,729	24,932	21,362	38,297
No. reads in contigs	132,714	157,011	155,924	100,079	545,728
No. singletons					7,775
No. unigenes					46,072
Mean length (bp)					1,100

Before assembly, the individual reads were tagged, where possible, relative to their putative sub-genomic origin based on Blat analysis. In total 20% of all reads were tagged as ‘A’ or ‘D’ (around 70,000 in each category). This low percentage may relate to the high similarity of both sub-genomes but also to an insufficient coverage of the 2 diploid reference transcriptomes. Reference-based assembly with MIRA using *Gossypium* EST assembly Cotton46 as a reference resulted in a unigene set of 46,072 sequences, 38,297 contigs (representing 545,728 reads) and 7,775 singletons (Table 1). Files containing the sequences and quality scores were deposited at the NCBIs (ref NCBI/SRA under accession numbers SRA051396.1).

**Figure 1:**
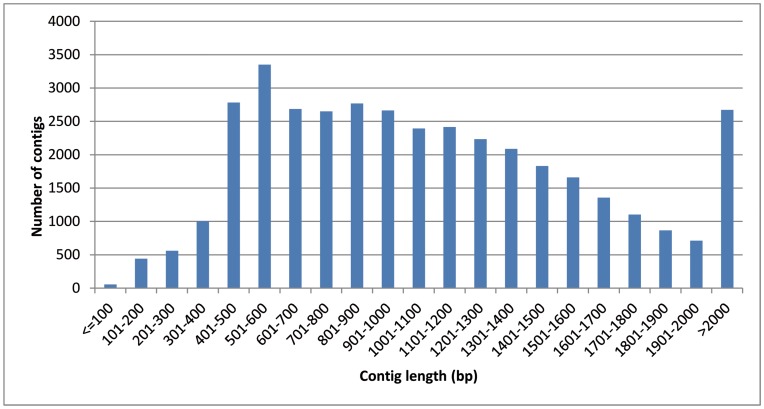
Length distribution (bp) of the 38,297 contigs of the global 454 assembly.

There were on average 14.2 reads per contig and 2% of the contigs had more than 100 reads (Table S1). Contig length was on average 1,100 bp with 6,933 bp as a maximum (Figure 1). Sixty two percent of all contigs were anchored to the contigs of the reference assembly Cotton46 (shown with a ‘bb’ tag) by MIRA; while 14,448 contigs (37%) were not anchored, suggesting that the single pyrosequencing run detected a substantial fraction of new fiber genes, thus providing deep coverage of the cotton fiber transcriptome. Conversely, 27% of the contigs in the reference assembly Cotton46 were not represented in our libraries. This may reflect the fact that the libraries used to assemble Cotton46 are derived from fiber as well as non-fiber tissues.

### Functional Annotation

**Figure 2:**
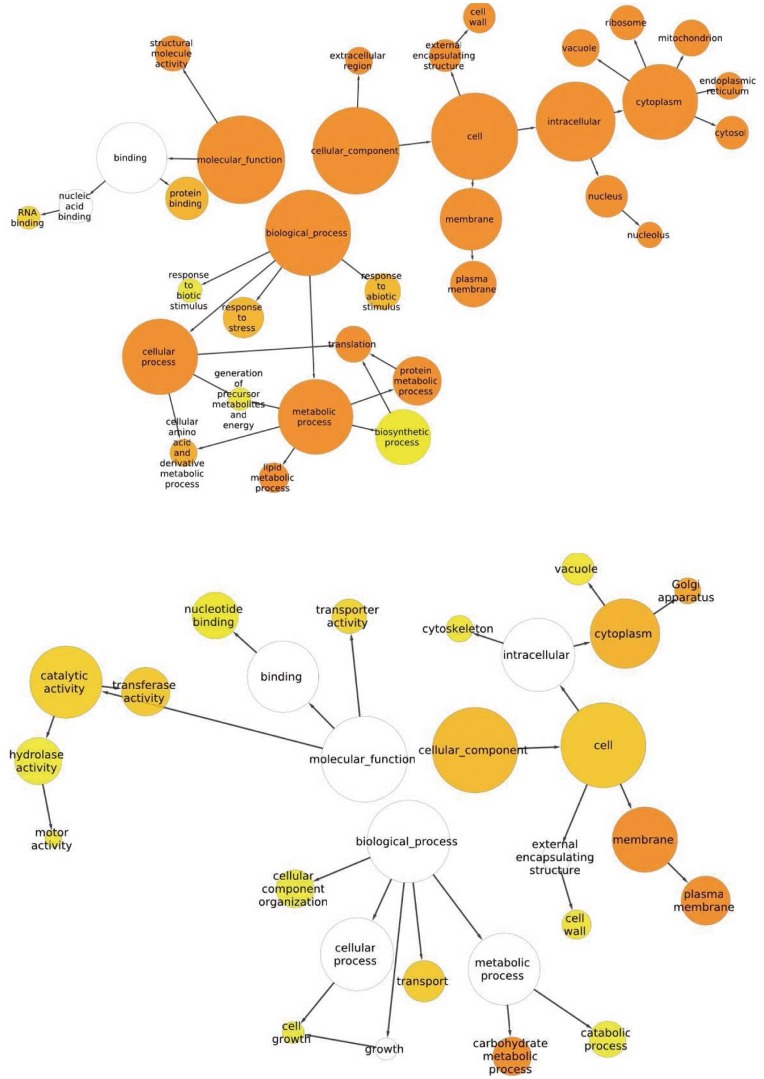
GO enrichment analysis between fiber development stages. The Gossip package of Blast2GO was used. Over representation of functional classes are presented among contigs with significant DGE (over-expression) at 10 compared to 22 dpa (upper Figure) and at 22 compared to 10 dpa (lower Figure). Significantly enriched GO terms (P<0.05) are highlighted, and the degree of color saturation of each node positively correlates with the enrichment significance of the corresponding GO term. Box color represent significance ranging from white for least significant to dark orange for most significant enrichment, arrows indicate hierarchical relationships.

The set of 38,297 contigs was Blast-searched against nr protein database using Blast2GO: 25,532 had an annotation and 2,669 contigs (7%) showed no similarity. Summaries of the functional categorization of contigs (89% were assigned a GO term) are shown in Figure S1 (full details tabulated in Table S2). The overall representation among the different functional GO terms was very similar to earlier reported global transcriptome data in plants, including cotton [35], with higher partitioning in the classes binding and catalytic activity (category molecular function), cellular and metabolic process (category biological process) and cell and organelle (category cellular component). The results of the GO enrichment analysis (performed in Blast2GO) between the two stages of fiber development are shown in Figure 2, for contigs showing differential DGE at 10 dpa (1,722 contigs) or at 22 dpa (1,048 contigs) relative to the other stage and irrespective of species of origin. Significantly more GO terms were enriched in the contigs over-represented at 10 dpa compared to those enriched at 22 dpa (Figure 2), consistent with the difference in the total number of contigs between these 2 categories. At 10 dpa, numerous GO terms were over-represented in the cellular component and biological process categories and to a lesser extent in the molecular function category, while at 22 dpa, enrichment was observed specifically for the carbohydrate metabolic process (the second most enriched term, plasma membrane, was also enriched at 10 dpa).

The comparison against the KEGG database [36] based on Blast2GO annotation algorithm, showed that 11,848 contigs (31%) had significant matches in the database with an enzyme commission (EC) number corresponding to 147 different KEGG pathways (Table S3).

#### Between-contig and within-contig annotation data

Some annotation redundancy (424 proteins had hits with 10 or more contigs) was observed, with for example high numbers of contigs annotated as alpha-tubulins (556 contigs), zinc finger proteins (320), or DNA-binding proteins (238). Among proteins with high representation were cellulose synthases (116) and lipid transfer proteins (116), both known to be important in fiber development. Redundancy may relate to: non-overlapping sequence reads from long transcripts (probably minimized in our case as we used a reference assembly), sequencing errors (454 sequencing technology is known to produce sequencing errors around homopolymer stretches), splice variants, allelism, or simply due to the fact that a number of genes occurred as different members (multigene families) such as paralogs and homoeologs.

The 10 most abundantly expressed genes (>1,000 reads) are presented in Table 2. Interestingly, the 1,363 reads annotated FbLate-2 protein exclusively originated from 22 dpa fibers (no read from the 10 dpa), with 3-times more from *Gb* than from *Gh* (1028 vs 335), consistent with previous reports [7] where FbL2A ranked first in terms of level of up-regulation in 24 dpa relative to 10 dpa fibers in *Gh*.

**Table 2:** Most highly abundant transcripts (contigs with >1,000 reads).

Contig	No. reads	*Gh*	*Gb*	10 dpa	22 dpa	Acc. Number*	Annotation
step1_rep_c47611	2,355	1,294	1,061	1,065	1,290	AAO92753.1	GhH6 (arabinogalactan protein 2)
step1_rep_c47637	2,242	894	1,348	706	1,536	ADB54351.2	cytochrome c oxidase
step1_rep_c47617	1,971	1,088	883	1,193	778	AAB03081.1	E6 (protein kinase)
step1_rep_c47607	1,363	335	1,028	0	1,363	AAA84881.1	FbLate-2**
step1_rep_c47612	1,357	859	498	1,019	338	AAL67991.1	dehydration-induced protein RD22-like protein
step1_rep_c47610	1,286	732	554	543	743	ABO47740.1	alpha-tubulin
step1_rep_c47608	1,221	502	719	940	281	AAG29777.1	lipid transfer protein 3 precursor
Contig_24629_bb	1,147	801	346	703	444	AAO18731.1	cysteine protease
step1_rep_c47623	1,096	349	747	420	676	ABO47736.1	alpha-tubulin
step1_rep_c47619	1,043	583	460	838	205	ABL86680.1	alpha-expansin

* Blastx were based on nr protein database; all accessions were from *Gossypium* species as ranked among 5 best hit scores except ADB54351 from *Allium.*

** the blastx annotation as FbLate-2 in *G. barbadense* ranked 11^t*h*^ (others were hypothetical protein), had 100% identity but only 25% coverage.

Number of reads of genotypes and fiber development stages are summed over 2 libraries each (*Gh* = Gh10+Gh22, *Gb* = Gb10+Gb22, 10 dpa = Gh10+Gb10, and 22 dpa = Gh22+Gb22).

#### Fiber expressed transcription factors

The 1,116 Gossypium hirsutum transcription factors, TF, present in the plant transcription factor database TFDB (http://planttfdb.cbi.pku.edu.cn/), representing 50 families, were used in a blastx search (E-value ≥10–20) of TFs among our contigs. As a result 1,710 contigs (or 4.4% of the 38,297 contigs) had a match with at least one sequence of the TF database (Table S4). Most TF families (49 out of 50) from the database were detected in our assembly, showing they are expressed in fibers. The 9 most abundant families, in terms of number of contigs, were MYB, MYB-related, bHLH, bZIP, C3H, Dof, NAC, C2H2, and ERF. Together they represented 48% of all TF annotations (Table S4) and were essentially similar in occurrence to those reported in fibers of G. hirsutum [12] or G. barbadense [35], or in other plant model species [35]. However, in terms of total representation (number of reads) the Dof family (DNA-binding with one zinc finger) was surprisingly abundant. The contigs assigned to the Dof TF class, which also included a majority of the actin annotations, had comparatively more reads per contig; and represented 20% of reads in the TF category (Table S4). Dof domain proteins are plant-specific TF with critical roles in growth and development [37], but have not yet been studied in any detail in cotton fiber.

#### Cell wall-related protein families expressed in fibers

**Figure 3:**
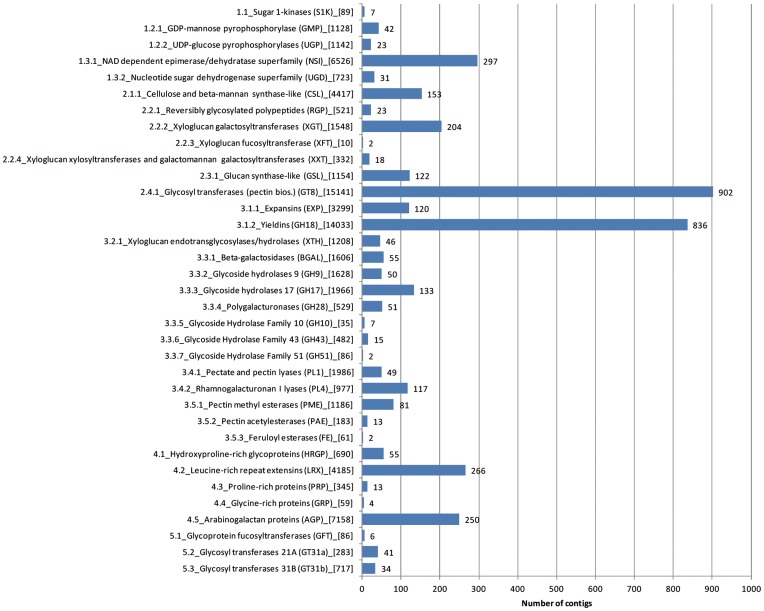
Number of contigs in the different cell wall-related gene categories. The gene categories definitions refer to the 2^nd^ or 3^rd^ organization levels in Cell Wall Navigator, http://bioweb.ucr.edu/Cellwall/). Number of reads are indicated in square brackets.

The database of plant cell wall biogenesis-related proteins, Cell Wall Navigator, CWN (http://bioweb.ucr.edu/Cellwall/) contains gene families that are involved in sugar substrate generation and primary cell wall (PCW) metabolism [38], with 4,591 sequences representing 35 major families. The blastx search among our contigs was based on a fairly low threshold (E-value ≥10–5) because the majority of sequences in CWN were not from Gossypium spp. (14% from Arabidopsis and from rice as major contributing species, and only 0.8% from Gossypium). 2,997 of our contigs (or 8%) had a homolog in the CWN database, corresponding to 4,070 hits (as some contigs hit sequences in multiple CWN categories) and 75,519 individual reads (or 14%) (Figure 3). The two most abundant cell wall categories were glycosyl transferases (category 2.4 with 22% of the cell wall-related contigs) and cell expansion (category 3.1, with 24% of the cell wall-related contigs), followed by nucleotide-sugar inter-conversion enzymes (category 1.3), AGPs (category 4.5) and glysosides hydrolases, including beta-galactosidases, BGAL, and glycoside hydrolases, GH (category 3.3). Within the cell expansion category the two sub-categories, expansins (3.1.1) and yieldins (3.1.2) represented 2.9% and 20.5% of cell wall-related contigs, respectively (Figure 3).

### Changes in Gene Expression

**Figure 4:**
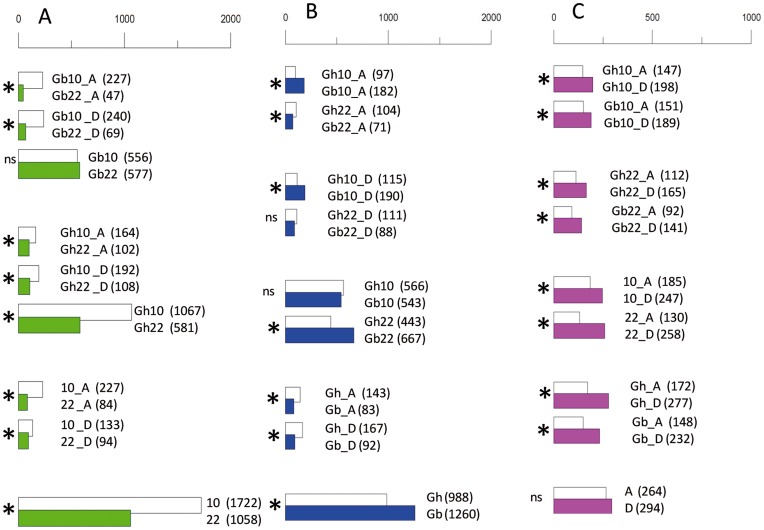
Changes in expression of fiber genes for all possible pair-wise comparisons. Comparisons are contrasted between: A- fiber development dates (10 vs. 22 dpa), B-genotypes (*G. hirsutum* and *G. barbadense*, *Gh* vs. *Gb*), and C-sub-genomic origin of reads (A sub-genome vs. D sub-genome). Gene expression was assessed digitally according to the origins of reads within contigs. In each pair-wise comparison bar length is proportional to the number of contigs showing differential over-expression (number of contigs also indicated in parentheses). Symbol ‘*’ indicates a statistical difference (P0.05) for each pair-wise comparison using Fisher’s exact test. For example, within the A panel (all comparisons involving 10 vs. 22 dpa), the comparison Gh10_A vs. Gh22_A (164 vs. 102, significantly different) indicates that within *G. hirsutum*, there are 164 contigs for which the A-tagged reads are more abundant (≥2-fold) at 10 dpa than at 22 dpa; conversely 102 contigs have more abundant representation of A-tagged reads at 22 dpa than at 10 dpa.

As the cDNA libraries used in this study were not normalized, differential DGE between libraries was assessed by read representation within contigs. In total, 4,828 different contigs were differential (fold ratio ≥2 and P<0.05) between genotypes, fiber development stages or sub-genomes (where known), or any combination of these 3 factors. All possible pair-wise comparisons are shown in Figure 4. The 289 most differentially-expressed genes (fold change ≥20) together with their blastx annotations against nr and *Gossypium-*specific protein databases are listed in Table S5.

Globally, more contigs showed an over-expression at 10 dpa as compared to 22 dpa (1,722 compared to 1,048, Figure 4A). This also holds true in *Gh* (1,067 for Gh10 compared to 581 for Gh22) but not in *Gb* (556 for Gb10 compared to 577 for Gb22). Some of the most interesting genes are presented below as either stage-preferential or genotype-preferential markers (presented in Table S5, including genes with higher representation and genes with fold ratio ≥20).

#### Marker genes of fiber development

Gene(s) (contigs) families displaying high differential expression in either the early or late stage of fiber elongation, irrespective of genotypes, are shown in Table 3. At the elongation stage these included ribosomal proteins (40S and 60S), LTPs, and expansins, while at the SCW stage these included beta tubulins, cellulose synthases and sucrose synthases. In addition, some differential contigs representing low-copy gene markers of the early stage included vacuolar invertase (VIN) (Contig_26940_bb) over-expressed in Gh10/Gh22, 3-ketoacyl-CoA reductase (step1_rep_c47717) over-expressed in 10/22 and an acyltransferase (Contig_36761_bb) (Table S5). Similarly, some differential gene markers of SCW (late stage) included 1,3-beta-glucanases (Contig_4209_bb), chitinase-like genes (Contig_9596_bb as 22>10 by 145-fold), FbLate-2 (Contig_46775_bb as 22>10 by 157-fold, Contig_44768_bb by 25-fold, and step1_rep_c47607 with 1363 reads exclusively from 22 dpa) and a Cobra-like4 protein (Contig_29232_bb as 22>10 by 75-fold) (Table S5).

**Table 3:** Genes most differentially expressed between the 2 stages of fiber development.

Gene annotation	Total contigs	No. contigs	No. contigs
		10 dpa vs 22 dpa	22 dpa vs 10 dpa
10>22 dpa			
60S ribosomal protein	307	68	1
40S ribosomal protein	200	49	0
Lipid transfer protein	119	11	1
GTP binding protein	92	11	0
proteasome	67	11	0
Histone (includes histone deacetylase	66	11	0
Alpha-expansin	65	8	0
proteasome (includes alpha and beta)	53	11	0
NADH ubiquinone oxidoreductase	41	9	1
14-3-3 protein	29	9	0
profilin	24	7	0
24-sterol c-methyltransferase	20	7	0
22>10 dpa			
beta tubulins (include annotation « beta chain »)	143	1	10
cellulose synthase	116	0	11
proline-rich protein	81	0	5
endosomal protein	40	1	10
vacuolar H^+^-translocating inorganic pyrophosphatase	36	0	6
sucrose synthase*	22	0	8

* Brill, et al. [72] reported 4 isoforms, A–D, ‘new ‘C isoform with highest level at SCW synthesis stage, which corresponded (best blast hits) to 2 of the contigs, Contig_19300_bb and Contig_56175_bb, which had the highest differential 22>10, with 67 and 103-fold.

#### Differential gene expression between genotypes

Consistent over-expression bias in one genotype relative to the other (irrespective of fiber development date) was observed in the case of Gb for genes coding for 60S ribosomal protein (52 contigs higher in Gb vs 1 higher in Gh), 40S ribosomal protein (all 40 higher in Gb), GTP binding protein (all 11 higher in Gb), proteasome (all 9 higher in Gb) and NADH ubiquinone oxidoreductase (all 6 higher in Gb). The opposite trend, i.e. over-expression in Gh, was observed for cellulose synthase (18 higher in Gh vs. 1 higher in Gb), sucrose synthase (all 8 higher in Gh), Cobra-like4 protein (3 contigs higher by 14 to 22-fold in Gh), and chitinase-like gene (Contig_33825_bb higher in Gh by 29-fold ) (Table S5). There were also 3 cases with an apparent interaction in terms of observed DGE between the combination of genotype and development stage: 1) Cobra-like4 protein, with 4 contigs all higher in Gh22 than Gb22; 2), white-brown complex ABC transporter, with 7 out of 8 contigs all higher in Gb22 than Gh22, and 3) ABC transporter, with 2 contigs (out of 3) displaying reverse trends in Gb and Gh, i.e. Gb22>Gb10 and Gh22<Gh10.

#### Differential fiber expression between homoeologs

The proportion of reads unambiguously tagged as ‘A’ or ‘D’ was around 20% (10% in each category), representing around 140,000 reads. Globally (Figure 4C), there was a slight, but not significant, bias in favor of an origin from the D sub-genome as compared to the A sub-genome (294/264). However, interestingly this trend in favor of the D sub-genome was found significant in all individual combinations, e.g. within genotypes and within fiber stages (Figure 4C). Contigs showing the most significant biases (>20 fold) in either sub-genome (26 contigs in each case) are listed in Table S5. The different cases where the A or D sub-genome biases were observed but in opposite directions with respect to development stage (32 contigs) or genotype (11 contigs) may represent cases of subfunctionalization between the two genomes [39], [40], and deserve further investigation but is beyond the scope of this article. Amongst the most interesting genes showing a sub-genome bias was the E6 protein kinase, represented by 12 contigs with A/D tags (7 with A-bias, none D-bias), including the 3 most A-biased contigs (Contig_41402_bb, step1_rep_c48769, step1_rep_c47674 and step1_rep_c47626 with more than a 40 fold expression difference between sub-genomes (Table S5).

### Affymetrix Expression Data and Comparison with DGE

Probe hybridization intensity (mean of 2 replicates) variation between fiber cDNAs at 10 dpa of *Gh* and *Gb* ranged between −10.5 and +7.8. In total, 3,697 genes displayed significant (Bonferroni test, P<0.05) differential intensities (Table S6), with no differences between the 2 genotypes in terms of numbers of genes up- and down-regulated (1,836 up-regulated in *Gb* and 1,861 up-regulated in *Gh*). Al-Ghazi, et al. [18] detected less (945) differentially expressed genes at a similar fiber development stage (11 dpa) using a 24K cDNA microarray, with different *Gh* and *Gb* genotypes (Siokra V-15 and Pima S7, respectively) and showed a slight bias in favor of up-regulated genes in *Gh* as compared to *Gb* (549 *Gh*+ vs. 396 *Gb*+). The annotations (Blast2GO) of our differentially regulated genes indicated that a significant number (75) coded for ribosomal proteins, a majority of which (84%) had higher expression in *Gb* than in *Gh* as was also observed through DGE (52 contigs showing a *Gb*-bias vs. only one showing a *Gh*-bias). Similarly, all but one of the 28 heat shock protein-annotated genes were highly expressed in *Gb* than in *Gh*. There were two chalcone synthase (CHS ) genes among genes most differentially over-expressed in *Gh*, in agreement with Al Ghazi, et al. [18]. The eleven differential (*Gh* vs. *Gb*) XETs were not consistently higher in one species or the other (8 were higher in *Gh* and 3 higher in *Gb*). Globally, the functional classification among GO terms of the 3,697 differentially expressed genes detected by Affymetrix arrays corresponded well with the classification of the 454 unigenes for this stage (not shown).

The 21,854 genes of the Cotton Affymetrix GeneChip® were BLASTed against the 454 unigene set (Cdhit, 90% similarity). Cross comparisons indicated that 9,979 contigs of the 454 unigene shared sequence similarity with at least one gene of the Affymetrix chip representing 2,915 different genes. Among the 543 contigs that were in common between the platforms and that showed significant differential expression between the 2 samples (eg 2 species at 10 dpa), 403 or 74% displayed the same direction of difference (242 genes with *Gh*>*Gb* in both platforms and 161 *Gb*>*Gh* in both platforms) while 140 showed a reverse sign (55 *Gb*-bias in 454 vs. a *Gh*-bias on the GenChip®; and 85 *Gh*-bias in 454 vs. a *Gb*-bias on the GenChip®). Overall the regression (R^2^ = 0.14) between expression ratios between species using the two platforms was not high, but was significant (P<0.05) (not shown) and may reflect environmental differences between the two locations in which the plants were grown.

### Detection and Validation of Gene-derived SSR Markers

The ESTs generated in this project also served as a resource to generate new gene-derived molecular markers. A total of 3,133 SSRs (2,930 different contigs containing 1 to 4 SSRs) with di- to hexa-nucleotides repeats, and compound motifs (Table 4) (3,889 homopolymeric repeats not shown) were identified. Dinucleotide repeats were identified in 1,016 cases, with the most frequent repeats being AT/TA (48%), AG/TC (40%) and AC/TG (12%); while the most frequent repeats among the 1,570 trinucleotide repeats were AAG/TTC (30%), AGT/ATC (12%), ACT/ATG (11%) and ACC/GGT (10%). Sequences of the 2,930 contigs were blastn-searched (e-value cutoff 1e-20) against existing cotton SSR resources in public databases (sequences retrieved from Cotton Marker Database at http://www.cottonmarker.org/and supplemented with some in-house CIRAD data, totaling 16,593 sequences). Under fairly stringent conditions, only 412 of the 2,528 SSR-containing contigs had no match with an existing sequence and hence are potentially novel. After trimming to eliminate short sequences and sequences with repeats positioned near the ends of contigs, 366 of these novel SSRs were further used to design primers and of those 339 successfully met all design criteria. The set of 339 putatively new and non-redundant EST-SSRs and their primers will be submitted to public cotton marker databases (CMD, CottonGen) under supplemental enumerations in the series “m*Gh*CIR”, i.e. m*Gh*CIR419 to m*Gh*CIR757 (summarized in Table S7). We selected a set of 88 of those SSRs (CIR419 to CIR506) for validation. All were successfully amplified in the 2 parents Guazuncho 2 (*Gh*) and VH8-4602 (*Gb*). Thirty three (38%) were polymorphic for at least one band between the parents and were mapped in the RIL genetic map [32]. Forty segregating loci were coded from 29 different EST-SSRs (11 segregated at 2 loci) and 35 new loci were integrated on the RIL genetic map using JoinMap (5 were unlinked) as detailed in Table S8.

**Table 4:** Summary table of SSR mining in the contigs.

SSR type	Total	Frequency per repeat motif (≥10%)
p2	1,016	AT (48%)-AG/CT (40%)-AC/GT (12%)
p3	1,570	AAG/CTT (30%)-AGT/ATC (12%)-ACT/ATG (11%)-ACC/GGT (11%)
p4	81	ACAT/ATGT (33%) - AAAT/TTTA (15%) - AAAC/GTTT (13%)
p5	21	AAGAG/CTCTT (14%)- AACTC/AGTTG (11%)
p6	112	ACTCGG/AGCCTG (25%) - AGGCTC/AGTCCG (23%)
compound	333	–
Total	3,133	

The frequencies of the different repeat motifs are presented in brackets.

### Detection and Validation of SNP Markers

The presence of sequence polymorphisms at a high frequency within contigs was clearly highlighted from visual alignments of 454 reads (2 examples shown in Figure S2). Overall, among 9,188 contigs exceeding a 6x depth threshold for at least one genotype were computationally mined for SNPs, 76,271 sequence variants were found in 6,931 different contigs. The 2 most variable contigs (205 and 162 SNPs) were both annotated as belonging to the multigene family of ubiquitin proteins. Among SNPs showing a bi-allelic pattern (99.7% of the total), transition mutations (A↔G, or T↔C) were the most frequent type (61%) as compared to transversion mutations (39%) as is commonly reported in plants [41], [42].

**Figure 5:**
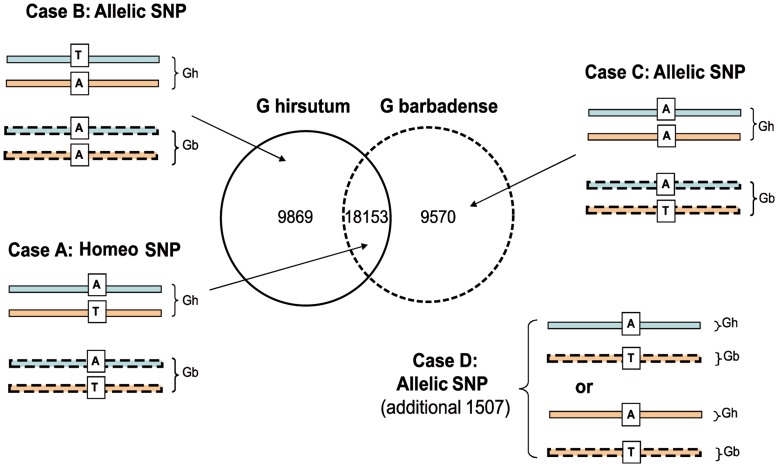
Schematic partitioning of the 39,099 variant positions between intra- and inter-genotypic SNPs. Variant positions are counted among contigs covered by the 2 genotypes and with at least 6 reads of both. The blue and brown bars symbolize the A_T_ and D_T_ sub-genome co-assembled homoeo-copies. Genotypes are symbolized in plain (*G. hirsutum*) and dotted (*G. barbadense*) lines.

Intra-genotype SNPs numbered 49,279 in *Gh* and 43,638 in *Gb*, corresponding to a density of 1 SNP every 82 and 79 bp in *Gh* and *Gb*, respectively. Such SNPs may either represent heterozygotes (expected to be low in our case as both genotypes are essentially inbred cultivars), or more likely to be variants between co-assembled genes, such as paralogous or homoeologous genes (i.e. sequence differences between genes of the 2 sub-genomes). The latter type could not be extensively and computationally verified [31], [41] as the rate of tagging of reads for their sub-genome origin was too low (<20%). However variation between homoeo-copies was visually confirmed for around 100 among the 753 contigs that had reads tagged for both sug-genome origins (example A in Figure S2). In a subsequent comparison, we focused only on positions covered with 6 or more reads of both genotypes (corresponding to 2,257,950 bp) and detected 39,099 SNPs (Figure 5). Among these SNP positions, 18,153 intra-genotypic SNPs were present within both genotypes (case A in Figure 5) and were interpreted as previously as polymorphisms between sub-genomes, or homoeo-SNPs. Another 19,439 polymorphisms occurred only in one genotype, *Gh* or in *Gb*, the other genotype being monomorphic (cases B and C in Figure 5). These polymorphisms represent inter-genotypic or allelic SNPs as they differentiate the 2 genotypes. The 39,099 SNPs also contained an additional 1,507 inter-genotypic polymorphisms that were monomorphic in both genotypes and for which it is assumed that only one of the sub-genomes was being expressed per genotype (case D in Figure 5). The overall density that we obtained for inter-genotypic SNPs differentiating our 2 genotypes (thus representing potential markers) was of 1 position every 108 bp (20,946 SNPs in total as the sum of cases B, C and D of Figure 5 from among 2.2 Mbp). All SNPs have been deposited in the SNiPlay database and are publically available at http://sniplay.cirad.fr, as well as in Table S9.

Forty eight triplets of primers (primer ‘F-Gua’, primer ‘F-VH8’ and primer ‘R’) covering a subset of the identified (allelic) inter-genotypic SNPs were synthesized for genotyping individuals from a RIL population [30] (Table S10). The 31 SNPs which amplified correctly and displayed a polymorphism for at least one band, resulted in 34 segregating bands (3 SNPs had 2 segregating bands). A majority of SNPs (28 of the 34 loci) were hemilogous (coded as dominant) where one parent has 2 alleles and the other has one allele and 6 were truly allelic (coded as co-dominant); the dominant-type of segregation is consistent with the proposed interpretation for inter-genotypic variations (case B in Figure 5). Thirty new EST-derived SNP loci were integrated into the RIL genetic map using JoinMap (4 were unlinked) (Table S8).

## Discussion

Advances in sequencing technologies now make it possible to conduct large-scale transcriptome analyses that can expand the catalogue of known expressed genes (particularly the less abundant genes) and simultaneously monitor the expression of tens of thousands of genes over time, different conditions, and in different tissues or in different genotypes. Prior to this study, the total number of EST sequences for *Gossypium* in GenBank (as of July 2011) had reached 5 million, including Sanger and 454 sequences (over 125 million when including Illumina short reads), although coverage of all species is not uniform. With the objective of increasing our knowledge of the fiber transcriptomes of the two main cultivated species *G. hirsutum* and *G. barbadense*, we sequenced cDNAs of developing fibers at two key developmental stages on a 454 sequencing platform. The four libraries (2 genotypes and 2 stages) generated 244 Mb of sequences that could be assembled into a unigene set of some 46,000 genes that contained over 14,000 new unigenes not present in the most extensive current *Gossypium* EST assembly, Cotton46 (http://cottonevolution.info/). Given that the number of genes in the cotton genome is estimated to be in the range 40–50,000 [43], it can be assumed that the assembled EST resources from this study (44,900 contigs in Cotton46, plus the 14,000 non-anchored contigs from our assembly) represent a substantial fraction of those genes, at least those expressed in developing fibers (75–94% of genes of the genome are thought to be expressed in cotton fibers [4]). Our data therefore represent an improvement both in terms of depth as well as of species representation of fiber ESTs, particularly for *G. barbadense*. Before this project, *Gb* was represented by less than 3% of Sanger sequences in Genbank even including a recent report [35] that added 10,000 sequences, and around 30% overall including all NGS reads. Although any analysis is yet to be published, a National Science Foundation project on comparative evolutionary genomics of cotton has also deposited a large amount of Illumina short read fiber EST sequences at 10 and 20 dpa, but for different genotypes of *Gh* and *Gb* (http://cottonevolution.info or http://trace.ncbi.nlm.nih.gov/Traces/sra/sra.cgi?study=SRP001603) which will be complementary to our data. Together these two studies will have improved the EST representation across a broader spectrum of fiber development time-points, particularly during the less-well documented SCW deposition stage. Indeed, the majority of earlier cotton EST libraries originated from ovules or fibers in the early stages of elongation.

### A Complex and Dynamic Gene Network during Cotton Fiber Development

Although only a quarter of a 454 plate was sequenced for each library, the depth of coverage achieved was sufficient to show the complexity of the fiber transcriptome, and the significant changes in transcription that occur during the switch from rapid fiber elongation to SCW deposition in both *Gossypium* species. As in previous studies [44] it was clear that a large proportion of the genome must be active during fiber growth even during SCW synthesis when almost a single biochemical process dominates the cell’s output – cellulose synthesis. Despite the greater coverage of genes afforded by the 454 platform relative to conventional Sanger EST sequencing, the molecular processes underlying the growth of the specialised fiber cell are still far from fully understood. A third or more of the contigs encode proteins that still are of unknown in function in any plant, and many have no corresponding matches to any plant genes and may thus be unique to cotton fibers. Annotation results, however, confirmed the timing and changes in abundance of many key fiber development genes that had previously been reported in the literature (e.g., [45]). A high representation of some gene families was evidenced by the large number of contigs with very similar annotations and by the number of reads within those contigs, such as tubulins [46]–[48], AGPs [49]–[51], E6 kinases [52]–[54], and actins [53], [55], [56] that are involved in the processes of PCW generation and expansion. Several highly abundant transcripts that were represented by more than 1000 reads in our assembly confirmed earlier reports of their high abundance in growing fibers. The protein kinase E6 (‘pCKE6’ in reference) was among the most abundant mRNA isolated from cotton fibers, and was detected throughout the development of the fiber [52], [54]. Its biological function still remains unclear, as no discernible phenotypic changes in fiber development were evidenced in transgenic cotton with reduced E6 protein levels [54]. Similarly, the proline-rich arabinogalactan protein H6 (‘pCK-H6’ in reference) that was amongst the first abundant proteins characterized in cotton fiber [57] was highly represented in our 454 assembly and in all 4 libraries. The FbL2A gene is a gene activated during late primary and early SCW synthesis stages in fiber, when massive quantities of cellulose are synthesized and deposited in the fiber [58]. It was highly abundant in the 22dpa stage (the 44 contigs annotated as FbLate-2 were all over-represented from 22 dpa libraries, ie 99% of the cumulative number of 3,800 total reads). However, the exact role of FbL2 also remains to be elucidated. Another abundant transcript in our assembly encoded an RD22-like protein (referred as RDL) that has been reported as showing a fiber-enriched expression pattern with no expression in other tissues [53].

Among all the unigenes, 1,710 (4.4%), representing nearly 22,000 reads, had a blast hit against an existing *Gossypium* putative transcription factor. The frequency of occurrence of TFs has been reported for 2 other cotton EST libraries to be 10% in *G. hirsutum* ovules [12] and 12.5% in developing fibers of *G. barbadense* [35], but in both cases those libraries were normalized prior to sequencing. All categories of the TF database were represented in our libraries, with a higher representation of MYB, MYB-related, bHLH, bZIP, C3H, Dof, NAC, C2H2, and ERF. Numerous MYB-related genes have been shown to regulate *Arabidopsis* leaf trichome as well as cotton fiber development, and have been the focus of intensive studies [45], [59]–[63].

The developing cotton fiber, a single cell of the ovule epidermis that can elongate up to 4–5 cm, represents a good model for the study of plant cell expansion and wall biogenesis. Among enzyme activities, many members of the cell wall-loosening expansin family are known to be expressed during fiber elongation, while cellulose synthesis is a predominant event in fiber cells in the SCW synthesis stage. In this context we focused our annotation analysis of the assembly against known cell wall-related proteins. The Cell Wall Navigator database was used to assign cell wall related annotations to 2,997 of the unigenes. Most cell wall-related genes in our EST libraries, as expected, were glycosyl transferases and cell expansion-related proteins, including expansins and yieldins. The central process in cell wall polysaccharide biosynthesis occurs through the action of a diverse array of glycosyltransferases [64], although many have still to be assigned an exact biosynthetic function. Yieldins, essentially represented in CWN by glycosyl hydrolases (other carbohydrate-acting enzymes) and chitinases, have been shown to participate in cell wall elongation in cowpea hypocotyls [65] and are likely to have a similar role in the extensive elongation of fiber cells.

### Comparison of 2 Gossypium Species Transcriptomes at the 2 Fiber Development Stages

We used the number of reads assembled in each specific contig to estimate digitally the expression of particular genes in the 2 genotypes and 2 development dates. We observed significant changes in gene expression between 10 dpa, at the peak of fiber elongation and 22 dpa, at the onset of SCW synthesis. Globally, the earlier stage was transcriptionally more complex than the later stage. The 10 dpa libraries contributed a higher number of contigs as compared to 22 dpa (33,750 vs. 30,658, +10%). This holds true also by the respective numbers of differential contigs at these two stages (Figure 4A), e.g. 1,722 contigs up-regulated at 10 dpa as compared to 1,058 at 22 dpa, and by the GO enrichment analysis (Figure 2) that showed many more ontology functional categories to be over-represented at 10 dpa. The observation that fiber elongation was a more active stage in terms of the abundance and diversity of transcripts confirms our recent report that nearly 3 times (3,263 vs. 1,201) more eQTLs were mapped at 10 dpa as compared to 22 dpa [34] in a fiber cDNA-AFLP experiment using a RIL population derived from the same two genotypes. Al Ghazi, et al. [18] noted that 20–30% more genes were more highly expressed during fiber elongation (11 dpa) than during SCW thickening (21 dpa) in their microarray analysis of two other cultivars of *Gb* and *Gh*. Recent Affymetrix-based hybridizations data reported twice more differentially expressed transcripts in 10 dpa-old fibers of *Gh* under drought stress as compared to 20 dpa (2,329 vs. 1,221) [9]. However, we also observed that the 10 dpa-bias was prevalent in *Gh*, with 1067 differential contigs for Gh10 and 581 for Gh22, and not in *Gb* (Figure 4B). This latter observation is possibly indicative of a time-lag difference in fiber development between the 2 species, whereby fibers of *Gb* would be transcriptionally active during a longer period as compared to *Gh*. Such hypothesis is supported in the genotypic pair-wise comparisons (*Gh* compared to *Gb* in Figure 4B) by the significantly higher number of over-expressed genes in *Gb* compared to *Gh* at 22 dpa (667 for Gb22 compared to 443 for Gh22) with no difference at 10 dpa (566 vs. 543). Other reports [66], [67] showed only minor differences between 10 and 20 dpa in fibers in 2 *Gh* accessions. Overall, the 454 data appear to provide a reliable representation of the transcriptomes of elongating and maturing cotton fibers, and in general, confirmed the involvement of known key enzymes and structural proteins at each of the different fiber stages. Apart from the cell wall-related genes mentioned above, such as expansin in the earlier stage and cellulose and sucrose synthases in the later stage; several other genes encoding enzymes involved in lipid metabolism were activated during fiber elongation, such as LTPs [68] or ketoacyl-CoA reductase [69]. This is consistent with the requirement in developing fiber cells for additional membrane synthesis, but also in signalling cascades involving lipids, particularly very long chain fatty acids [70] (Table S5). In the later stage, apart from the accumulation of a series of well described cell wall-related genes such as tubulins, cellulose and sucrose synthases [71], [72] and invertase [10], [73]–[75], we observed differential expressions for 1,3-beta-glucanase [16], [18], [76], [77], chitinase-like gene [4], [35], [51], [78], [79], and a Cobra-like4 protein [51], [80] (Table S5). Among the 116 contigs annotated as cellulose synthases, 11 showed differential expression relative to fiber development stage (Table 3). In all cases expression at 22 dpa was higher that at 10 dpa. This observation is not completely in agreement with that of Kim and Triplett [71] who showed that among 14 putative CesA isoforms, some were expressed early in elongating fibers, while others were expressed during SCW thickening or throughout fiber development.Transcription factors with differential expression according to development stage (117 contigs among 2,770, or 4.2%) were equally shared between contigs up-regulated at 10 dpa (59 contigs) and at 22 dpa (58 contigs), unlike an earlier report [12] suggesting an up-regulation of TFs mainly during early stages of fiber and ovule development.

As previously mentioned the two species studied have significant differences in fiber quality at maturity, with the *Gb* variety having fibers that are around 33% longer, 31% stronger and 29% finer than those in the *Gh* variety [32], [33]. It was expected that the magnitude of the phenotypic differences, that are considered extremely large in terms of cotton breeding objectives, would also be reflected in significant differences at the transcriptional level during fiber development. A large number of genes (2,248 contigs, or 6% among the 38,297) were differentially expressed between the varieties irrespective of the stage of fiber development. Such gene expression variation between *Gh* and *Gb* fibers had been reported in Al Ghazi, et al. [18] where differential expression between *Gh* and *Gb* decreased from approximately 19% (4513 genes differential among 24,000 cDNA probes) at 7 dpa, to 4% (945 genes) at 11 dpa and 1% (258 genes) at 21 dpa. In the same study, pathways related to secondary metabolism and pectin synthesis were amongst the most differentially expressed between *Gh* and *Gb* during the peak elongation stage. Although annotations with secondary metabolism were abundant in a number of contigs in our assembly, we did not observe such a genotypic difference in our material in these GO categories. Different cellulose synthases were noted to be quite stage-specific with high abundance at the SCW stage and completely absent from the 10 dpa samples (at 22 dpa, the number of reads of *CesA1*, *CesA2* and *CesA7* in *Gh*/*Gb* were 620/59, 383/30 and 415/22 respectively, with none in either species at 10 dpa). Expression of these SCW-specific *CesA*s was considerably lower in the Gb22 library. These results on *CesA*s are illustrative of the overall difference between *Gh* and *Gb* in the number of contigs showing differential DGE at 10 dpa and at 22 dpa, with significantly more differential genes in Gh10 compared to Gh22 and no difference between Gb10 and Gb22 (Figure 4B). It is possible that the two genotypes under the conditions used here are not as synchronous in their progression through fiber development as the genotypes in Al Ghazi, et al. [18], and that the onset of SCW deposition is more delayed in the *Gb* genotype such that a prolonged elongation period may account for its longer and finer fibers.

Although limited to the elongation stage (10 dpa), the Affymetrix hybridizations confirmed the genotypic differences in expression between *Gh* and *Gb*. A first evidence of reproducibility between the 2 techniques was provided by the fact that Blast2GO categories corresponded well. Among the 543 genes in common between the Affymetrix chip and the 454 unigene and that showed differential expression, 403 (74%) followed a similar direction on the 2 platforms, including 5 LTPs, 6 alpha- and 3 beta-tubulins, and 2 endo-1-4-beta glucanases, all over-expressed in *Gh* (Table S6).

### The D-genome may have a Greater Contribution to Fiber Development than Expected

In (allo)polyploids duplicated gene copies are partly free from functional constraints and can evolve different evolutionary paths, from pseudogenization, to neo- or sub-functionalization [39], [40]. Since part of the 454 reads were tagged for their putative sub-genomic origin, we were able to investigate sub-genomic expression bias at least in those genes for which we could identify both homoeologous copies. Interestingly, a preference for transcription from the D_T_ sub-genome homoeolog was observed within genotypes and within fiber development stages (Figure 4C). In terms of fiber development stage, twice as many contigs were D_T_-genome biased at 22 dpa as compared to 10 dpa. This agrees with Hovav, et al. [66] who used microarray hybridization to compare expression of different homoeologs. They examined expression of 1,500 pairs (A_T_- and D_T_-genome homoeologs) of homoeologous genes, and showed that homoeolog contributions were unequal with an overall bias towards D_T_-genome transcription throughout fiber development, but particularly later in development. In our cDNA-AFLP study [34] eQTLs at 10 dpa had a slight A-bias with respect to their chromosome localization (1,925 on A_T_ chromosomes vs. 1,740 on D_T_ chromosomes) while the reverse held true at 22 dpa (627 vs. 748). These transcriptome-based data emphasizing the contribution of the D_T_-genome in fiber development tetraploid cotton support mapping experiments of QTLs for fiber quality traits showing that both A_T_ and D_T_ chromosomes from tetraploid cotton hosted QTLs and metaQTLs [33], [81]. Modern A-genome diploid species produce spinnable fibers while D-genome species do not. Assuming that the same remark could stand for the A and D ancestor diploids of tetraploids, it could be that the A-sub-genome harbored favorable fiber alleles prior to polyploid formation, whereas the D-sub-genome would have come under selection after polyploid formation and contributed new allelic diversity within the domesticated tetraploid forms [82].

### More Gene-based Markers for Marker-assisted Selection in Cotton Breeding

Large-scale sequencing of ESTs has been a source of a considerable number of gene-based markers, including EST-SSRs which are now widely used in constructing linkage maps, diversity analysis, etc. in many crop species including cotton (see different cotton SSR projects reviewed in Cotton Marker Database, CMD, at http://www.cottonmarker.org/): more than half of the 18,000 public *Gossypium* SSRs gathered so far in CMD were derived from EST-SSRs. Gene-derived SNPs are also important sources of useful molecular markers, especially in cotton where cDNA sequencing has been extensive across a diverse array of germplasm sources, although they have been used in only two instances for genetic mapping [41], [83]. In this study, 339 new EST-SSR and close to 20,000 inter-genotypic polymorphisms (allelic SNPs), represented among 3,150 different contigs, have been generated. Although it is expected that only a proportion of these markers will be informative amongst diverse germplasm, they have extended the repertoire of genic markers available in cotton for various purposes. Sixty five new loci (35 SSRs and 30 SNPs) designed from gene-based markers have enriched the genetic map that we had previously developed for our RIL population.

### Conclusion

In this research we describe a large-scale transcriptome analysis from fibers of the two cultivated cotton species, *G. hirsutum* and *G. barbadense*. An EST collection has been generated by 454 pyro-sequencing of cDNA from the 2 species at 2 fiber development stages, 10 and 22 days post anthesis. The assembled unigene set of 46,000 sequences (38,300 contigs and 7,700 singletons) has expanded the catalogue of genes expressed during fiber elongation. As compared to EST resources presently in Genbanks, the representation has been improved for the under-represented species *G. barbadense* (as compared to *G. hirsutum* and to diploid genomes) and for fibers in a more advanced stage of development (22 dpa corresponding to transition from primary to secondary cell wall synthesis). Comparative analysis of digitally-based and hybridization-based expression data has provided evidence for large differences in the abundance of transcripts for key genes involved in a number of metabolic pathways potentially controlling fiber development, and thus potentially involved in explaining the contrasting fiber characteristics of the 2 species.

## Materials and Methods

All new data reported in this study had been deposited to Genebank (see text for accessions numbers).

### Plant Material

cDNA libraries for sequencing were constructed from developing fibers from the two genotypes, Guazuncho 2 (*G. hirsutum* L.) and VH8-4602 (*G. barbadense* L.), used as parents for different inter-specific mapping populations including backcross (BC) and recombinant inbred line (RIL) populations [32]. Four plants of each genotype, further referred as *Gh* and *Gb*, were grown under glasshouse conditions in Montpellier (France) as earlier described [34]. Flowers were tagged on the day of anthesis. Boll locules (containing seeds and fibers) from 2–5 bolls per plant were collected at two dates, 10 or 22 dpa, between 10.00 am and 12.00 am and immediately frozen in liquid nitrogen and stored at −80°C till use. The two fiber developmental time points were chosen as key stages corresponding to the phase of peak fiber elongation (10 dpa) and rapid SCW synthesis stage (22 dpa).

### cDNA Preparation and Sequencing

Four fiber cDNA libraries representing the two genotypes and two stages of fiber development, were constructed from total RNA extracted from pooled fiber samples of several bolls from two plants as earlier described [34] following the protocol based on MATAB and LiCl precipitation. RNA quality was checked on a 1.4% denaturing agarose gel. Total nucleic acids were quantified by UV absorbance (DU530, Beckman Coulter, Fullerton, CA, USA) and DNA contaminations were quantified by fluorescence of the dye Hoechst 33258 with a Fluoroskan fluorimeter (Ascent, Labsystems, Finland). Samples were DNaseI-treated, followed by a phenol/chloroform extraction and a precipitation with Na-acetate and ethanol. RNAs were resuspended in water and 5 µg of RNAs were sent to GATC Biotech (Constanz, Germany, Inc.) for cDNA sequencing, (http://www.gatc-biotech.com/en/sequencing/transcriptomes.html ) without a normalization step, on a 454 GS FLX™ Titanium genome sequencer (Roche, Inc.). A quarter-plate sequencing run was performed for each of the four libraries (referred as Gh10, Gb10, Gh22 and Gb22) according to GATC Biotech proprietary methods.

### EST Data Processing

#### Editing, trimming and assembly

Prior to their assembly reads were labeled where possible according to their sub-genome of origin by comparing each read against two separate EST assemblies of the A and D sub-genomes that constitute the polyploid cotton genome. ESTs (Sanger-only) from G. arboreum (Ga, A sub-genome progenitor) and G. raimondii (Gr, D sub-genome progenitor) were retrieved from Genbank and assembled separately (CAP3, 95%, 100 bp overlap), resulting in 6,469 contigs and 19,898 singletons for Ga and 8,650 contigs and 15,768 singletons for Gr. All reads were compared using Blat software [84] against the two assemblies using default parameters. Reads were assigned an ‘A’ or ‘D’ tag depending on the results of the Blat. Tags with equal similarity against both progenitors or with similarity with only one progenitor were labelled as unknown sub-genome. This approach has been used previously and has allowed the A and D copies to be assessed as separate entities, including in terms of relative expression between homoeologs [66].

A global (all four libraries pooled) reference-based assembly was made using MIRA software (default parameters for this type of assembly) [85]. We used the public data of hybrid Sanger/454 cotton ESTs assembly Cotton46 from http://cottonevolution.info/as reference.

#### Digital gene expression analysis

The comparison of the number of EST reads (normalized to the overall number of reads in each of the libraries) within contigs was used as an indicator of the relative digital gene expression, DGE, and for comparing different libraries. To access the differentially expressed genes we used the R statistic described in Sketel et al. [86] and we accepted the genes with p-value less than 0.05 and fold change greater than 2. In our case, pairwise comparisons were made possible between genotypes (irrespective of fiber stage), fiber development stages (irrespective of genotypes), and A/D sub-genomes, as well as for all combinations of these factors, such as for example the relative representation of A vs. D tags within the Gh genotype at 10 dpa.

#### Functional annotation

Gene ontology (GO) annotation of the contigs (singletons not considered) were assigned using Blast2GO [87], [88] using a minimum E-value cutoff of 1e-6. We also retrieved the EC numbers and constructed KEGG pathways [36] based on the Blast2GO annotation algorithm. GO enrichment analysis was implemented in the GOSSIP package of Blast2GO using Fisher’s exact test (alpha = 0.05). Statistically enriched functional classes were identified between the two stages of fiber development, 10 and 22 dpa using the two sets of contigs showing differential DGE between the two conditions, either over-expressed at 10 or at 22 dpa, respectively.

Additionally, two targeted functional classifications were undertaken to identify putative (i) cell wall-related proteins and (ii) transcription factors. The Cell Wall Navigator, CWN [38] database was used to retrieve sequences (4,767) of proteins that are involved in plant cell wall biogenesis. Secondly *Gossypium* transcription factors were retrieved from the Plant Transcription Factor DataBase, Plant TFDB (1,116 *Gossypium* TFs present as protein sequences downloadable at http://planttfdb.cbi.pku.edu.cn/). The nucleotide sequences of the 454 contigs were then blastx-searched against both protein sequences.

### Microsatellite and SNP Identification

Identification of microsatellites, or Simple Sequence Repeats (SSR), was performed using the MISA search tool [89] specifying a minimum of 10 (mononucleotide), 6 (di-nucleotides motifs) and 5 repeats (tri- to hexa-nucleotides), and a maximum of 100 bases interruption for compound repeats. Primers for detection of the SSRs were designed using BatchPrimer3 [90].

Putative SNPs were mined from the global assembly of 454 cDNA sequences originating from the 2 genotypes. Due to the potential co-assembly of copies from the constituent sub-genomes of a polyploid genome, it is expected that different types of variants would be recovered, such as true allelic variants between genotypes, paralogs and heterozygotes, but also variants between copies of the individual sub-genomes (also referred as homoeo-SNPs) [31]. In the case of tetraploid AD cotton, the presence of homoeologous variants from the A_T_ and D_T_ sub-genomes has long been recognized and diagnosed through sequence alignment with sequence resources from contemporary diploid A and D species [41]. SNP mining was based on bio-informatic pipelines developed locally, including a dedicated module for SNP discovery from Ace assemblies such as those provided by the MIRA assembler. Resulting SNPs, consensus alleles and sequences were finally integrated in the SNiPlay database [91] (http://sniplay.cirad.fr/). SNiPlay includes a searchable Web interface where all SNPs can be queried using different criteria. Searches for sequence variants only considered base changes (indels not considered), and was conducted at 2 levels, within genotype and between genotypes. At the intra-genotypic level, SNPs were called independently on the 2 genotypes if the following conditions were met: (1) coverage depth of the given genotype was ≥6, and (2) the minor allele occurred in at least 10% of the alleles examined and with a minimum of 2 occurrences. At the inter-genotypic level, only positions respecting the 6x coverage depth for the 2 genotypes (i.e. 12x minimum) were considered.

### Validation of SSRs and SNPs

We selected 88 SSRs for validation using DNAs from the two genotypes. Polymorphic SSRs were then screened over DNAs of the RIL population (140 individuals) derived from the same two genotypes following our standard protocol [32]. Segregating bands/loci were then integrated on the RIL genetic map using JoinMap as previously described [32].

From the computationally mined putative inter-genotypic SNPs, 100 were visually confirmed using Tablet [92], and 48 were selected based upon their clear allelic partitioning between the two sequenced genotypes and secondly on their amenity for designing diagnostic PCR primers. The protocol described in Gaudet, et al. [93] was then followed with minor modifications for genotyping the RIL mapping population by allele-specific PCR and electrophoresis. The two forward allele-specific primers for the 2 genotypes were tailed with 5 or 15 bases (to make a 10 bp size difference in the resulting PCR product), respectively, and 2 different destabilizing mismatches were introduced at position −4 at the 3′ end. Primers were designed with Primer 3 (http://primer3.sourceforge.net/). Oligo Analyzer V3.1 (http://www.idtdna.com/analyzer/applications/oligoanalyzer/Default.aspx) was then used to optimize the three primers reactions (less hairpin loops and duplexes) and primer design was revised when necessary. Genotyping and mapping conditions of SNPs were similar to SSRs, except that the PCR was based on a touch-down with a T_m_ from 65°C to 58°C (with a step of 3°C).

### Affymetrix Hybridizations and Expression Data

Plants from the two parental genotypes, Guazuncho 2 and VH8-4602 were cultivated under glasshouse conditions in Canberra, Australia. Total RNA was extracted from 11 dpa-old fibers of the 2 genotypes with two independent replicates of each, using the protocol from Wan and Wilkins [94]. Pooled RNA from 4 plants was checked for quality and quantity using an Agilent Bioanalyser 2100 (Agilent Technologies, Santa Clara, CA, USA, http://www.home.agilent.com) following the manufacturer’s recommendations. The RNA was sent to the Australian Genome Research Facility Ltd. (http://www.agrf.org.au, Melbourne, Victoria, Australia) for labeling and hybridization to the Affymetrix Genechip® Cotton Genome Array (21,854 genes) (Affymetrix, http://www.affymetrix.com/). The data were normalized with the gcrma algorithm [95], available in the Bioconductor package [96]. To determine differentially expressed genes, we performed a two group t-test that assumes equal variance between groups. The variance of the gene expression per group is a homoscedastic variance, where genes displaying extremes of variance (too small or too large) were excluded. The raw P values were adjusted by the Bonferroni method, which controls the Family Wise Error Rate (FWER) [97]. A gene was declared differentially expressed if the Bonferroni P-Value was less than 0.05. All these steps were performed on an Affymetrix analysis pipeline at INRA-URGV, Evry, France. The raw.CEL files were imported in R software for data analysis. All raw and normalized data are available through the CATdb database (AFFY_COTTON_2011_12, [98]]) and from the Gene Expression Omnibus (GEO) repository at the National Center for Biotechnology Information (NCBI) [99], accession number GEO:GSE36876.

## Supporting Information

Figure S1 **Gene Ontology classification of the global 454 cDNA assembly.** The level 2 of classification was used. Pooled data from 2 genotypes and 2 fiber development dates were analyzed. The number of contigs is indicated in parentheses.
(TIF)

Figure S2 **Two exemplary contigs (Tablet screenshots) displaying the presence of SNPs. A-** Example of intra-genotypic SNPs in Contig_46738_bb (75 reads, 1770 bp). Shown is region 509–560 bp with 8 homoeo-SNPs: reads at all 8 positions are unambiguously tagged as belonging to the A and to the D sub-genomes respectively: - 515 bp (allele T for A the sub-genome/allele C for the D sub-genome), - 521 bp (G/A), - 524 (C/T), - 530 (A/G), - 535 (T/G), - 544 (C/T), - 547 (T/C), and –556 (A/G). **B**- Example of inter-genotypic SNPs in Contig_5257_bb (191 reads, 1750 bp). Shown is region 406–462 bp with one SNP between the 2 genotypes in position 423 (G/A): all reads with allele G originate from *Gb* and all reads with allele A originate from *Gh.*
(DOC)

Table S1 Distribution of the number of reads in unigenes.
(DOC)

Table S2 Results of the level 2 and 3 GO term assignment by Blast2GO.
(XLS)

Table S3 Ten most represented KEGG pathways (number of sequences).
(XLS)

Table S4 Frequency of the putative transcription factor categories found among the 38,297 contigs.
(DOC)

Table S5 List of 289 contigs with highest digital differential expression. The values higher than 20-fold expression change are highlighted in yellow color. Differential expression is calculated among either of 7 pairwise comparisons: Gb10/Gb22, Gb10/G10, Gb22/Gh22, Gh10/Gh22, Gb/Gh, 10/22 or Ga/Gr (Gb = *G. barbadense*, Gh, *G. hirsutum*, Ga = *G. arboreum* of A-genome, and Gr = *G. raimondii* of D-genome; 10 and 22 refer to fiber development stages in days post anthesis, dpa). The blastx annotations are made against both unspecified non redundant protein database and *Gossypium* taxid as database.
(DOC)

Table S6 List of 3,697 differential genes detected from Affymetrix hybridizations. Comparison between Guazuncho 2 (*Gh*) and VH8-4602 (*Gb*) and for fibers at 10 dpa: mean intensities over 2 replicates and Blast2GO annotation.
(XLS)

Table S7 Primer sequences of the 339 new non redundant EST-SSRs (series m*Gh*CIR419 to m*Gh*CIR757).
(XLS)

Table S8 Mapping results of EST-SSR and EST-SNP markers. Chromosome localization of segregating loci are indicated (“unl” indicates an unlinked locus in JoinMap software).
(DOC)

Table S9 Sequences of the 39,099 inter-genotypic SNPs. SNPs are representing allelic polymorphisms between Guazuncho 2 (*G. hirsutum*) and VH8-4602 (*G. barbadense*). Sequences are provided in the Illumina submission format, with 60 flanking bp on each side of SNP. SNP names include contig name in the assembly and SNP position in the unpadded alignment.
(XLS)

Table S10 Sequences of the 48 triplets of primers used for SNP genotyping, including SNP name as used in mapping, Contig id, product size as from EST assembly (may differ from PCR product size from genomic DNA).
(XLS)
