# Supplementary figures and images for: Deep Sequencing Reveals Differences in the Transcriptional Landscapes of Fibers from Two Cultivated Species of Cotton
Source: PLoS One. 2012 Nov 15;7(11):e48855. doi: 10.1371/journal.pone.0048855 (PMC3499527; doi:10.1371/journal.pone.0048855)

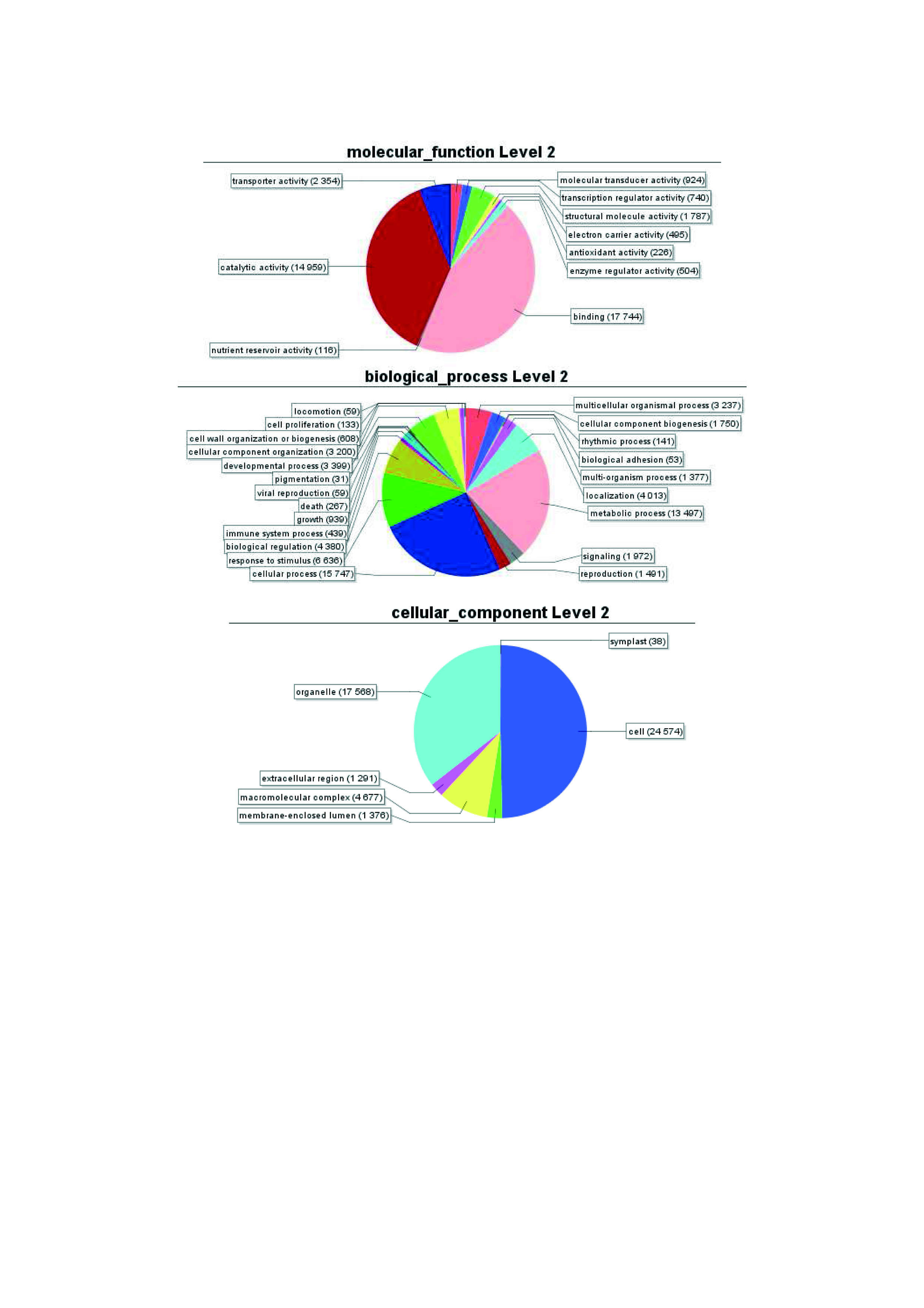

Supplement: Figure S1 — Gene Ontology classification of the global 454 cDNA assembly. The level 2 of classification was used. Pooled data from 2 genotypes and 2 fiber development dates were analyzed. The number of contigs is indicated in parentheses. (TIF) [file pone.0048855.s001.tif]
