# Supplementary material for: Deep Sequencing Reveals Differences in the Transcriptional Landscapes of Fibers from Two Cultivated Species of Cotton
Source: PLoS One. 2012 Nov 15;7(11):e48855. doi: 10.1371/journal.pone.0048855 (PMC3499527; doi:10.1371/journal.pone.0048855)
Supplement: Figure S2 — Two exemplary contigs (Tablet screenshots) displaying the presence of SNPs. A- Example of intra-genotypic SNPs in Contig_46738_bb (75 reads, 1770 bp). Shown is region 509–560 bp with 8 homoeo-SNPs: reads at all 8 positions are unambiguously tagged as belonging to the A and to the D sub-genomes respectively: - 515 bp (allele T for A the sub-genome/allele C for the D sub-genome), - 521 bp (G/A), - 524 (C/T), - 530 (A/G), - 535 (T/G), - 544 (C/T), - 547 (T/C), and –556 (A/G). B- Example of inter-genotypic SNPs in Contig_5257_bb (191 reads, 1750 bp). Shown is region 406–462 bp with one SNP between the 2 genotypes in position 423 (G/A): all reads with allele G originate from Gb and all reads with allele A originate from Gh. (DOC) [file pone.0048855.s002.doc]

**Figure S2 : Partial views (Tablet screenshots) of 2 exemplary contigs displaying the presence of SNPs**

1. Example of intra-genotypic SNPs in Contig_46738_bb (75 reads, 1770 bp). Shown is region 509-560 bp with 8 homoeo-SNPs: reads at all 8 shown positions are unambiguously tagged as belonging to the A and to the D sub-genomes respectively: - 515 bp (allele T for A sub-genome/allele C for D sub-genome), - 521 bp (G/A), - 524 (C/T), - 530 (A/G), - 535 (T/G), - 544 (C/T), - 547 (T/C), and – 556 (A/G)

1. Example of inter-genotypic SNPs in Contig_5257_bb (191 reads, 1750 bp). Shown is region 406-462 bp with one SNP between the 2 genotypes in position 423 (G/A): all reads with allele G originate from *Gb* and all reads with allele A originate from *Gh*
