# Supplementary material for: Deep Sequencing Reveals Differences in the Transcriptional Landscapes of Fibers from Two Cultivated Species of Cotton
Source: PLoS One. 2012 Nov 15;7(11):e48855. doi: 10.1371/journal.pone.0048855 (PMC3499527; doi:10.1371/journal.pone.0048855)
Supplement: Table S1 — Distribution of the number of reads in unigenes. (DOC) [file pone.0048855.s003.doc]

**Table S1: Distribution of the number of reads in unigenes**

| No. reads | No. unigenes |
| --- | --- |
| 1 | 7,775 |
| 2 | 10,921 |
| 3 | 5,438 |
| 4 | 3,524 |
| 5 | 2,510 |
| 6 | 1,817 |
| 7 | 1,523 |
| 8 | 1,241 |
| 9 | 980 |
| 10 | 836 |
| 11-20 | 4,413 |
| 21-30 | 1,729 |
| 31-40 | 954 |
| 41-50 | 554 |
| 51-60 | 332 |
| 61-70 | 282 |
| 71-80 | 201 |
| 81-90 | 139 |
| 91-100 | 104 |
| >100 | 799 |
| Total | 46,072 |
