# Supplementary material for: Deep Sequencing Reveals Differences in the Transcriptional Landscapes of Fibers from Two Cultivated Species of Cotton
Source: PLoS One. 2012 Nov 15;7(11):e48855. doi: 10.1371/journal.pone.0048855 (PMC3499527; doi:10.1371/journal.pone.0048855)
Supplement: Table S4 — Frequency of the putative transcription factor categories found among the 38,297 contigs. (DOC) [file pone.0048855.s006.doc]

**Table S4: Frequency of the putative transcription factor categories found among the 38,297 contigs**

| Category | No. Contigs | % | No. reads | % |
| --- | --- | --- | --- | --- |
| MYB_related | 145 | 8.3 | 1,836 | 8.6 |
| bHLH | 120 | 6.8 | 985 | 4.6 |
| MYB | 104 | 5.9 | 1,120 | 5.3 |
| bZIP | 92 | 5.2 | 762 | 3.6 |
| C3H | 88 | 5.0 | 1,049 | 4.9 |
| Dof | 79 | 4.5 | 4,363 | 20.5 |
| NAC | 78 | 4.4 | 1,785 | 8.4 |
| C2H2 | 77 | 4.4 | 815 | 3.8 |
| ERF | 65 | 3.7 | 686 | 3.2 |
| B3 | 63 | 3.6 | 409 | 1.9 |
| HD-ZIP | 59 | 3.4 | 640 | 3.0 |
| GRAS | 54 | 3.1 | 406 | 1.9 |
| Trihelix | 54 | 3.1 | 374 | 1.8 |
| G2-like | 47 | 2.7 | 285 | 1.3 |
| WRKY | 46 | 2.6 | 575 | 2.7 |
| ARF | 43 | 2.5 | 293 | 1.4 |
| HB-other | 43 | 2.5 | 502 | 2.4 |
| TALE | 43 | 2.5 | 407 | 1.9 |
| TCP | 39 | 2.2 | 399 | 1.9 |
| FAR1 | 35 | 2.0 | 182 | 0.9 |
| MIKC | 34 | 1.9 | 770 | 3.6 |
| GATA | 26 | 1.5 | 150 | 0.7 |
| DBB | 25 | 1.4 | 216 | 1.0 |
| HSF | 25 | 1.4 | 117 | 0.5 |
| AP2 | 24 | 1.4 | 194 | 0.9 |
| S1Fa-like | 23 | 1.3 | 379 | 1.8 |
| SBP | 18 | 1.0 | 64 | 0.3 |
| CO-like | 17 | 1.0 | 144 | 0.7 |
| ARR-B | 16 | 0.9 | 83 | 0.4 |
| GRF | 14 | 0.8 | 42 | 0.2 |
| NF-YB | 14 | 0.8 | 100 | 0.5 |
| BES1 | 13 | 0.7 | 121 | 0.6 |
| EIL | 13 | 0.7 | 238 | 1.1 |
| LBD | 13 | 0.7 | 113 | 0.5 |
| ZF-HD | 13 | 0.7 | 63 | 0.3 |
| BBR/BPC | 11 | 0.6 | 56 | 0.3 |
| NF-YC | 11 | 0.6 | 56 | 0.3 |
| GeBP | 9 | 0.5 | 187 | 0.9 |
| NF-YA | 9 | 0.5 | 52 | 0.2 |
| Nin-like | 9 | 0.5 | 27 | 0.1 |
| VOZ | 8 | 0.5 | 68 | 0.3 |
| E2F/DP | 6 | 0.3 | 19 | 0.1 |
| Whirly | 6 | 0.3 | 66 | 0.3 |
| WOX | 6 | 0.3 | 22 | 0.1 |
| SRS | 5 | 0.3 | 24 | 0.1 |
| CPP | 4 | 0.2 | 13 | 0.1 |
| CAMTA | 3 | 0.2 | 13 | 0.1 |
| RAV | 3 | 0.2 | 11 | 0.1 |
| M-type | 2 | 0.1 | 4 | 0.0 |
| Total | 1,754 |  | 21,285 |  |
