# Supplementary material for: Deep Sequencing Reveals Differences in the Transcriptional Landscapes of Fibers from Two Cultivated Species of Cotton
Source: PLoS One. 2012 Nov 15;7(11):e48855. doi: 10.1371/journal.pone.0048855 (PMC3499527; doi:10.1371/journal.pone.0048855)
Supplement: Table S5 — List of 289 contigs with highest digital differential expression. The values higher than 20-fold expression change are highlighted in yellow color. Differential expression is calculated among either of 7 pairwise comparisons: Gb10/Gb22, Gb10/G10, Gb22/Gh22, Gh10/Gh22, Gb/Gh, 10/22 or Ga/Gr (Gb = G. barbadense, Gh, G. hirsutum, Ga = G. arboreum of A-genome, and Gr = G. raimondii of D-genome; 10 and 22 refer to fiber development stages in days post anthesis, dpa). The blastx annotations are made against both unspecified non redundant protein database and Gossypium taxid as database. (DOC) [file pone.0048855.s007.doc]

**Table S5: List of 289 contigs with highest digital differential expression.** Values higher than 20-fold expression change between 2 given situations are highlighted in yellow color. Seven pairwise comparisons are considered: Gb10/Gb22, Gb10/G10, Gb22/Gh22, Gh10/Gh22, Gb/Gh, 10/22 or Ga/Gr (*Gb=G barbadense, Gh, G hirsutum, Ga= G arboreum* of A-genome, and *Gr= G raimondii* of D-genome ; 10 and 22 refer to fiber development stages in days post anthesis, dpa). The blast annotations are made against both unspecified non redundant protein database and *Gossypium* taxid as database. Contigs with similar *Gossypium* blastx annotation are grouped.

| **Contig** | **Gb10/ Gb22** | **Gb10/ Gh10** | **Gb22/ Gh22** | **Gh10/ Gh22** | **Gb/**  **Gh** | **10/**  **22** | **Ga/**  **Gr** | **Seq. Description (Blast2Go nr)** | **Manual blastx (*Gossypium* taxid)** | **Reference** |
| --- | --- | --- | --- | --- | --- | --- | --- | --- | --- | --- |
| Contig_24216_bb |  |  | -4,28 | -47,02 | -7,14 | -81,32 |  | protein | 1,3-beta-glucanase cov7% max35% | [1,2,3,4,5] |
| Contig_44280_bb | 1,03 | 1,23 | 2,49 | 2,09 | 1,71 | 1,52 | -23,85 | 14-3-3 protein | 14-3-3e protein | [6,7,8,9] |
| step1_rep_c47717 | 8,35 | -1,84 |  | - | -1,15 | 32,21 |  | steroid | 3-ketoacyl-CoA reductase 1 | [3,10,11] |
| Contig_44505_bb | -22,97 |  | 9,52 | -1,47 | 4,17 | -7,64 | -4,90 | at1g68530 t26j14_10 |  |  |
| Contig_38327_bb | 1,76 | 1,32 | 22,33 | 29,82 | 2,47 | 4,10 | 2,09 | hydroxymethylglutaryl- synthase |  |  |
| Contig_22396_bb | 2,65 | 4,10 | - | - | 7,39 | 5,03 | 30,05 | beta ketoacyl-coa synthase family protein |  |  |
| Contig_45369_bb |  |  |  |  |  | -20,34 |  | amp dependent | 4-coumarate:CoA ligase 1 | [3] |
| Contig_42281_bb | -2,17 | -2,76 | 16,36 | 20,77 | 1,12 | 2,05 | 8,10 | white-brown-complex abc transporter family | ABC transporter | [12,13] |
| Contig_52455_bb | 1,57 | -1,24 | 22,33 | 43,38 | 1,59 | 4,51 |  | white-brown-complex abc transporter family |  |  |
| Contig_6247_bb | -2,23 | -1,11 | 29,24 | 14,51 | 2,76 | 1,24 |  | white-brown-complex abc transporter family |  |  |
| Contig_23498_bb | 2,78 | -2,10 |  | 32,53 | -1,22 | 8,65 | -9,39 | aldo keto | acetohydroxyacid synthase | [14] |
| step1_rep_c48031 | 7,04 | -1,24 |  | 4,90 | 1,01 | 5,05 | 39,64 | actin | Actin | [7,15,16] |
| step1_rep_c47694 |  | -9,83 | -1,82 | 1,20 | -4,02 | -1,46 | -25,02 | actin depolymerizing factor 2 | actin depolymerizing factor 3 | [17,18] |
| Contig_44133_bb | 13,91 | 9,52 |  |  | 14,82 | 24,16 | 16,62 | hydroxycinnamoyl shikimate quinate hydroxycinnamoyltransferase | acyltransferase-like protein | [7,15] |
| Contig_55199_bb | 8,19 | 14,98 |  |  | 23,93 | 13,74 |  | hydroxycinnamoyl shikimate quinate hydroxycinnamoyltransferase |  |  |
| Contig_28096_bb | 15,26 | - |  |  | - | 24,39 |  | hydroxycinnamoyl- shikimate quinate hydroxycinnamoyltransferase |  |  |
| Contig_56303_bb | - |  | -1,47 | -24,24 | -2,49 | -50,84 |  | transferase family protein |  |  |
| Contig_13416_bb | 8,09 | 1,32 |  | - | 2,10 | 20,42 | -17,21 | transposon protein mutator sub-class |  |  |
| Contig_35872_bb | 1,23 | 1,08 | - | - | 2,34 | 3,36 | 32,82 | anthranilate n-benzoyltransferase |  |  |
| Contig_31085_bb | -1,67 | -1,50 | 3,04 | 2,72 | 1,28 | 1,27 | -26,20 | adenosine nucleotide translocator | adenine nucleotide translocator 1 |  |
| Contig_55535_bb | 1,67 | 1,43 |  |  | 1,75 | 2,04 | 29,41 | adp-ribosylation factor | ADP-ribosylation factor | [19] |
| Contig_27599_bb | - | -1,92 |  | 4,31 | -1,69 | 4,73 | -21,11 | agamous-like protein | agamous-like protein 2 [Gossypium barbadense] MADS-box protein MADS4 |  |
| Contig_54722_bb |  | -44,79 | -1,61 | 1,93 | -5,67 | -1,03 | -1,03 | aluminum-induced protein | Al-induced protein |  |
| Contig_24592_bb |  |  | -11,36 | -77,87 | -18,81 | -125,38 |  | at1g19870 f6f9_18 | alpha-1,4 glucan phosphorylase cov10% max25% |  |
| step1_rep_c48885 | 3,52 | -1,66 |  | 32,53 | 1,00 | 9,53 | 1,66 | alpha expansin | alpha-expansin | [4,20,21,22,23] |
| Contig_5341_bb |  | - |  | 28,46 | - | 18,79 |  | aspartyl protease family protein |  |  |
| Contig_12955_bb |  | - |  | 48,79 | - | 32,21 |  | aspartyl protease family protein |  |  |
| Contig_28608_bb | -5,40 |  | -4,07 | -20,94 | -5,19 | -16,16 | - | tubulin alpha 3 | alpha-tubulin | [22,24,25] |
| step1_rep_c47663 | 2,55 | 1,35 | -1,71 | 1,11 | 1,10 | 1,52 | 28,77 | potassium channel beta subunit | aquaporin PIP | [26] |
| Contig_46043_bb | -32,54 | -17,12 | -9,34 | -17,75 | -15,13 | -26,75 |  | plasma membrane intrinsic protein |  |  |
| Contig_16119_bb | 3,70 | -1,58 |  | 32,53 | 1,05 | 9,75 |  | protein | benzoquinone reductase | [27,28] |
| Contig_38936_bb | 7,21 | 1,98 |  | 54,21 | 3,11 | 14,20 | - | beta galactosidase 11 | beta-galactosidase | [29,30] |
| Contig_17156_bb |  |  | -3,43 | -23,51 | -3,05 | -7,58 |  | beta galactosidase expressed |  |  |
| Contig_38042_bb |  |  | -3,15 | -38,94 | -4,75 | -35,02 |  | beta chain | beta-tubulin | [3,7,29] |
| Contig_54935_bb | -32,39 | -16,39 | -2,92 | -5,76 | -5,11 | -9,68 | -5,21 | beta chain |  |  |
| Contig_15569_bb | -37,97 | -26,35 | -2,42 | -3,49 | -4,59 | -6,23 |  | beta chain |  |  |
| Contig_11988_bb | -3,30 |  | 20,46 |  | 2,42 | -1,04 |  | beta tubulin |  |  |
| step1_rep_c47760 |  | - | -2,94 | -2,49 | -6,26 | -4,51 | -23,46 | tubulin beta 4 chain |  |  |
| Contig_48605_bb | -83,17 |  | -6,93 | -218,56 | -11,17 | -241,35 |  | beta chain |  |  |
| Contig_32046_bb | - |  | 1,18 | -13,96 | -1,47 | -35,02 |  | lysine-ketoglutarate reductase saccharopine dehydrogenase bifunctional enzyme | bifunctional lysine-ketoglutarate reductase/saccharopine dehydrogenase |  |
| Contig_40547_bb | -27,00 |  | -6,84 | - | -10,53 | -233,26 |  | cellulose synthase | cellulose synthase 2 | [21,29,31,32] |
| Contig_41561_bb |  | -2,81 |  | 20,33 | -2,01 | 19,68 |  | aldo keto |  |  |
| Contig_34451_bb | 3,05 | 1,12 |  | 20,33 | 1,85 | 6,57 | 7,67 | aldo keto |  |  |
| step1_rep_c47640 |  |  | -10,08 | - | -14,19 | -134,42 |  | cellulose synthase |  |  |
| Contig_19481_bb |  |  | -16,06 | - | -26,37 | - |  | cellulose synthase | cellulose synthase catalytic subunit | [21,29,31] |
| Contig_22861_bb | -3,96 |  | -6,33 | -95,50 | -7,20 | -26,46 |  | cellulose synthase |  |  |
| step1_rep_c47739 |  |  | -15,57 | -21,31 | -26,37 | -33,88 |  | cellulose synthase |  |  |
| step1_rep_c47657 | - |  | -3,60 | -54,36 | -5,99 | -96,02 |  | PREDICTED: hypothetical protein [Vitis vinifera] |  |  |
| step1_rep_c47836 |  |  | -21,47 | - | -35,16 | - |  | cellulose synthase |  |  |
| Contig_14067_bb |  |  | -49,38 | -8,45 | -43,95 | -11,65 |  | lysine histidine transporter |  |  |
| Contig_52992_bb | 5,55 | 1,52 | 13,08 | 47,67 | 2,43 | 11,64 | -1,18 | chalcone isomerase | chalcone isomerase | [3] |
| Contig_32865_bb |  | - | -11,81 | 2,85 | -59,75 | 1,77 | -3,22 | chalcone synthase | chalcone synthase | [3,33] |
| Contig_33825_bb |  |  | -17,93 | - | -29,44 | - |  | at3g16920 k14a17_4 | chitinase-like protein | [30,31,34,35,36] |
| Contig_9596_bb | - |  | -3,53 | -82,28 | -5,84 | -145,72 |  | at3g16920 k14a17_4 |  |  |
| Contig_43091_bb |  | -22,39 |  | 11,58 | -3,34 | 2,66 | 3,84 | protein | cinnamoyl-CoA reductase 1 | [3] |
| step1_rep_c48163 | - |  | 27,90 |  | 17,10 | - |  | hypothetical chloroplast rf15 | class III peroxidase | [37] |
| Contig_53061_bb | -1,03 | -1,06 | 22,33 | 23,04 | 2,08 | 2,59 |  | auxin-responsive family protein |  |  |
| Contig_29232_bb |  |  | -11,25 | -46,28 | -18,75 | -74,56 |  | cobra-like 4 protein | COBRA-like 4 protein (no Gossypium) | [35,38] |
| Contig_53608_bb | -1,17 | 1,30 | 20,46 |  | 2,90 | 2,00 |  | cytochrome b5 | copper binding protein 5 [Gossypium hirsutum] cov13% | [39] |
| step1_rep_c49099 | 10,57 | 14,47 |  |  | 22,80 | 17,89 |  | cysteine protease cp5 | cysteine protease [Gossypium hirsutum] cov9% | [40] |
| Contig_53317_bb | 2,22 | 1,39 | -1,02 | 1,58 | 1,39 | 1,90 | -38,32 | protein |  |  |
| step1_rep_c48043 | - | 1,52 |  | 10,21 | 2,01 | 19,96 | 40,92 | cytosolic ascorbate peroxidase | cytosolic ascorbate peroxidase 1 [Gossypium hirsutum] | [7,41] |
| Contig_47435_bb | 3,55 | 1,52 |  | 21,79 | 2,48 | 7,09 |  | 21 kda protein | DELLA protein GAI [Gossypium barbadense] cov24% max27% |  |
| Contig_29243_bb |  | -48,74 |  | - | -32,53 | - |  | delta tonoplast intrinsic protein | delta-tonoplast intrinsic protein | [42] |
| Contig_49929_bb | 40,62 | - |  |  | - | 65,30 |  | protein | dirigent-like protein 2 |  |
| Contig_41402_bb | -6,69 | -1,69 | -1,12 | -4,45 | -1,69 | -5,67 | 147,04 | 5 start site is | E6 | [15,43,44] |
| step1_rep_c48769 | 10,57 | 4,82 |  |  | 2,84 | 3,25 | 33,24 | protein |  |  |
| step1_rep_c47674 | -16,65 | -14,42 | 1,33 | 1,15 | -2,06 | -2,09 | 42,19 | 5 start site is |  |  |
| step1_rep_c47626 | -1,30 | -1,37 | 1,30 | 1,38 | -1,06 | 1,04 | 59,07 | 5 start site is |  |  |
| Contig_55128_bb | 8,33 | -2,01 |  | - | -1,26 | 33,70 | - | endo-beta -glucanase | endo-1,4-beta-glucanase [Gossypium hirsutum] | [4,45] |
| Contig_51273_bb | 2,46 | 11,81 |  |  | 10,83 | 3,25 | -31,28 | protein |  |  |
| Contig_10813_bb | -36,16 | -15,80 | 1,17 | -1,96 | -1,84 | -4,51 |  | protein |  |  |
| Contig_5709_bb |  |  | -6,13 | -15,13 | -10,55 | -25,20 |  | beta xylosidase alpha l-arabinosidase |  |  |
| Contig_35921_bb | -3,60 | -4,12 | 26,04 | 29,82 | 1,04 | 1,72 |  | phenylalanine ammonia- | fasciclin-like arabinogalactan protein 13 | [35,46,47] |
| Contig_17671_bb | -20,70 | -10,49 | 3,58 | 1,81 | 1,02 | -2,19 |  | beta ig-h3 domain-containing protein |  |  |
| Contig_6280_bb |  |  | -5,41 | -22,28 | -7,51 | -22,50 |  | beta ig-h3 domain-containing protein |  |  |
| Contig_26578_bb | - |  | -6,14 | -202,03 | -10,11 | -337,74 |  | beta ig-h3 domain-containing protein |  |  |
| step1_rep_c47845 |  | - | -6,98 | 1,47 | -23,74 | -1,13 |  | cell adhesion protein |  |  |
| step1_rep_c47690 |  |  | -20,93 | - | -34,28 | - |  | fasciclin-like arabinogalactan protein 5 |  |  |
| Contig_54593_bb |  |  | -17,18 | - | -28,12 | - |  | fasciclin and related adhesion glycoprotein |  |  |
| Contig_32984_bb |  |  | - | -13,96 | - | -21,46 |  | endosomal p24a protein | Fb35 | [48] |
| step1_rep_c47986 |  |  | -6,25 | -25,72 | -3,52 | -6,11 |  | coatomer protein epsilon subunit family protein cope family protein | FbLate-2 * | [29,31,49] |
| Contig_46775_bb | -25,31 |  | -4,78 | - | -7,32 | -157,00 |  | protein |  |  |
| Contig_44568_bb |  |  | -2,41 | - | -3,17 | -24,86 |  | protein |  |  |
| step1_rep_c47624 |  |  | -41,03 | - | -67,32 | - |  | ---NA--- |  |  |
| Contig_47161_bb |  |  | -4,71 | -32,33 | -7,91 | -55,34 |  | myb-like dna-binding domain containing protein | fiber dTDP-glucose 4-6-dehydratase | [29] |
| Contig_15996_bb |  |  | -6,83 | -37,47 | -5,71 | -12,38 |  | udp-glucuronic acid decarboxylase 2 |  |  |
| step1_rep_c47765 |  |  | 1,40 | -8,82 | -1,27 | -23,72 |  | sp1l5 (spiral1-like5) | fiber glycosyl hydrolase family 19 protein | [50] |
| Contig_55364_bb | 7,30 | 35,03 | -1,07 | -5,14 | 7,03 | 3,97 |  | heat shock | fiber protein |  |
| Contig_33744_bb |  |  | -3,88 | -21,31 | -6,59 | -37,28 |  | protein |  |  |
| Contig_15377_bb |  |  | -7,50 | -15,43 | -12,89 | -25,42 |  | protein |  |  |
| step1_rep_c47693 | 1,41 | 3,55 | 20,46 |  | 6,33 | 2,51 |  | sugar binding |  |  |
| Contig_39698_bb |  |  | 1,12 | - | -1,32 | -27,10 |  | zinc finger family protein |  |  |
| Contig_38192_bb | 2,00 | -1,90 |  | 35,24 | -1,03 | 6,50 |  | at1g04360 |  |  |
| step1_rep_c48032 |  | -1,97 |  | - | -1,20 | 22,38 |  | rbx1 (ring-box 1) protein binding |  |  |
| Contig_56332_bb |  | -2,76 |  | 28,46 | -1,02 | 2,75 |  | protein |  |  |
| Contig_40542_bb | -4,29 | -2,19 | 23,34 | 11,90 | 2,07 | -1,09 |  | l-asparaginase |  |  |
| Contig_38939_bb |  | - |  | 19,07 | -26,37 | 8,30 |  | nodulin 3 family protein |  |  |
| Contig_38503_bb | 2,78 | 1,59 |  | 2,04 | 1,71 | 2,53 | -23,46 | mitochondrial f1- gamma subunit |  |  |
| Contig_21693_bb |  | -5,62 |  | 20,43 | -2,16 | 4,10 | 2,56 | flavanone 3-hydroxylase | flavanone-3-hydroxylase | [12] |
| Contig_50934_bb |  | -4,72 |  | 24,39 | -2,78 | 10,20 |  | gip1-like protein | GASA-like protein [Gossypium hirsutum] cov29% |  |
| Contig_41775_bb |  | -2,81 |  | 20,33 | -1,41 | 4,88 |  | aux iaa protein | Gbiaa-Re |  |
| Contig_11966_bb |  |  | - | - | -27,25 | -35,02 |  | 2-oxoglutarate-dependent dioxygenase | gibberellin 3-hydroxylase 1 | [31,51] |
| Contig_47838_bb | 1,59 | -3,28 |  | 34,05 | -1,69 | 6,90 | -21,90 | glutamine synthetase | glutamine synthase | [52] |
| Contig_35714_bb |  | - |  | 24,39 | -5,57 | 3,99 |  | glutamine synthetase |  |  |
| Contig_54256_bb |  |  | -2,34 | - | -3,42 | -48,58 |  | glutathione s- | glutathione S-transferase | [12] |
| Contig_41180_bb | 21,64 | 2,12 |  | 38,13 | 3,14 | 31,33 | -4,01 | glutathione s-transferase |  |  |
| Contig_44654_bb | 1,18 | -4,71 |  | 82,67 | -2,18 | 7,69 | 1,24 | dehydroascorbate reductase |  |  |
| Contig_1931_bb | -26,44 |  | 2,57 | -2,61 | 1,30 | -7,14 | 3,62 | glycerol-3-phosphate o-acyltransferase | glycerol-3-phosphate o-acyltransferase |  |
| Contig_52802_bb | 2,36 | 1,85 | -1,74 | -1,37 | 1,20 | 1,25 | 21,74 | tpl protein binding protein homodimerization transcription repressor | glycine-rich RNA-binding protein |  |
| Contig_31989_bb | -21,70 |  | -1,78 | -5,88 | -3,04 | -9,76 | - | granule-bound starch synthase | granule-bound starch synthase 1 |  |
| step1_rep_c47916 | -21,70 | -18,44 | 4,48 | 3,81 | -1,29 | -1,28 |  | ---NA--- | H6-like protein | [53] |
| Contig_43633_bb | 3,89 | 21,33 |  |  | 12,15 | 4,29 |  | 40s ribosomal protein s6 |  |  |
| Contig_40966_bb | 1,18 | 12,95 |  |  | 28,50 | 2,00 |  | icr4_arath ame: full=interactor of constitutive active rops 4 |  |  |
| Contig_43896_bb | -21,03 | -2,85 | -1,72 | -12,72 | -2,75 | -17,50 | -2,80 | proline-rich cell wall protein |  |  |
| step1_rep_c47679 | -2,19 | -1,69 | 3,18 | 2,45 | 1,26 | 1,05 | 33,24 | heat shock protein 70 | heat shock protein 70 | [8,15] |
| step1_rep_c48265 | 1,67 | -1,19 |  | 25,75 | 1,59 | 4,43 | 2,11 | heat shock protein 70 |  |  |
| Contig_39536_bb |  | -35,57 |  | 18,39 | -8,50 | 6,21 |  | homeobox-leucine zipper protein hat7 | homeobox protein |  |
| Contig_34479_bb | -1,10 | -1,60 | 1,21 | 1,76 | -1,18 | 1,27 | -27,37 | nadp-specific isocitrate dehydrogenase | Isocitrate dehydrogenase |  |
| Contig_35512_bb | -1,47 | -1,56 | 2,57 | 2,72 | 1,16 | 1,38 | -24,24 | 3-isopropylmalate dehydrogenase |  |  |
| Contig_27397_bb |  |  | -2,44 | -30,12 | -2,84 | -11,25 |  | kinesin-like protein | kinesin-like calmodulin binding protein | [54,55,56] |
| Contig_45947_bb | 1,18 | -1,56 | 27,90 | 51,50 | 1,37 | 3,88 |  | kinesin-like protein |  |  |
| Contig_44873_bb |  |  | -9,66 | -13,23 | -16,70 | -21,46 |  | kinesin-like protein | kinesin-related protein |  |
| Contig_22965_bb | - | 2,39 |  |  | 3,13 | 25,95 |  | leucine aminopeptidase | leucine aminopeptidase |  |
| Contig_27344_bb | -13,50 |  | 27,90 |  | 3,22 | -2,58 |  | leucine-rich repeat family protein | leucine-rich repeat resistance protein-like protein |  |
| Contig_37372_bb |  |  | -17,18 | - | -28,12 | - |  | protein |  |  |
| Contig_43112_bb |  |  | -3,29 | -31,59 | -5,52 | -56,48 |  | protein | LIM domain protein | [3] |
| Contig_16850_bb | 4,89 | 2,10 |  | 21,68 | 3,28 | 8,87 |  | alpha l-fucosidase 2 | lipase |  |
| Contig_11659_bb | 4,36 | -1,00 | 13,08 | 57,20 | 1,64 | 10,75 | 1,68 | gdsl-motif lipase hydrolase family protein |  |  |
| Contig_39556_bb | 1,61 | -7,83 | -4,66 | 2,71 | -5,86 | 1,85 | -31,28 | zinc finger |  |  |
| Contig_23544_bb | - | 1,25 |  | 44,72 | 1,81 | 77,84 |  | zinc finger |  |  |
| Contig_9135_bb | -21,60 |  | -1,65 | -7,77 | -2,74 | -12,27 |  | zinc finger |  |  |
| Contig_26841_bb |  |  | -4,39 | -60,24 | -7,30 | -103,92 |  | lipid binding | lipid binding protein, putative [Ricinus communis] | [36] |
| step1_rep_c49117 |  | -7,69 |  | 27,92 | -5,40 | 21,28 |  | lipid transfer protein | lipid transfer protein precursor | [22,29,54,57] |
| step1_rep_c48116 |  | -6,00 |  | 43,38 | -3,63 | 17,29 |  | lipid transfer protein |  |  |
| Contig_56254_bb | -1,20 | -3,79 | 2,35 | 7,46 | -1,80 | 2,90 | -30,50 | lipid transfer protein |  |  |
| step1_rep_c47759 | - | -2,08 |  | 51,50 | -1,43 | 55,46 | - | lipid transfer protein |  |  |
| Contig_39312_bb | 6,74 | 64,74 |  |  | 10,47 | 4,77 | 1,55 | kda class ii heat shock protein | low molecular weight heat shock protein |  |
| Contig_30434_bb |  | - |  | 20,33 | - | 13,43 |  | major latex | major latex-like protein | [29] |
| Contig_18698_bb | -61,48 |  | -1,56 | -36,37 | -2,54 | -50,08 | 3,84 | proton-dependent oligopeptide transport family protein | mannitol transporter |  |
| Contig_31630_bb | -9,00 |  | -1,39 | - | -1,90 | -20,33 |  | proton-dependent oligopeptide transport family protein |  |  |
| Contig_49231_bb | 1,94 | -14,35 |  | 20,82 | -7,96 | 10,58 | -6,35 | shikimate kinase family protein | metallothionein-like protein | [58] |
| Contig_31820_bb | 12,80 | 8,76 |  |  | 13,68 | 22,38 |  | r2r3-myb transcription factor | myb transcription factor | [59,60,61] |
| Contig_51016_bb |  | -32,94 |  | 11,33 | -12,31 | 5,77 |  | nucleoside transporter |  |  |
| Contig_53851_bb |  |  | -17,13 | - | -28,12 | - |  | protein |  |  |
| Contig_23308_bb | 34,50 | 1,28 |  | 16,77 | 1,79 | 21,95 |  | acid phosphatase class b family protein |  |  |
| Contig_9756_bb |  |  | -11,81 | -8,08 | -21,09 | -12,99 |  | nac domain ipr003441 | NAC domain protein NAC5 | [62] |
| Contig_28573_bb |  | -35,57 |  | 9,19 | -27,25 | 6,21 |  | nodulin 21 family protein | nodulin family protein | [29] |
| Contig_46001_bb | -3,34 |  | 24,18 |  | 2,53 | -1,05 |  | protein |  |  |
| Contig_14136_bb | 2,17 | 2,11 | 22,33 | 23,04 | 3,73 | 4,37 |  | cytochrome p450 | P450 monooxygenase | [29] |
| Contig_35596_bb |  | -5,24 |  | 21,68 | -1,50 | 2,54 |  | pectate lyase | pectate lyase | [63] |
| Contig_38188_bb | -1,43 | -2,38 | 85,57 | 142,31 | 1,12 | 3,08 | -79,77 | pectate lyase |  |  |
| step1_rep_c47691 |  | -7,66 |  | 47,43 | -2,43 | 4,54 |  | pectate lyase family protein |  |  |
| Contig_5100_bb |  |  | - | -18,00 | - | -27,67 |  | pentatricopeptide repeat-containing | pentatricopeptide repeat protein | [64] |
| Contig_49976_bb |  | - | -8,05 | 3,54 | -47,46 | 2,16 | 1,39 | secretory peroxidase | peroxidase | [37] |
| Contig_32033_bb |  | -32,13 | -2,86 | 4,17 | -11,43 | 2,38 | 3,61 | secretory peroxidase |  |  |
| Contig_2700_bb |  | -2,33 |  | 21,68 | -1,25 | 5,54 |  | multidrug pheromone mdr abc transporter family | P-glycoprotein | [29] |
| step1_rep_c47646 | -1,76 | -3,52 | -1,25 | 1,61 | -2,20 | 1,01 | -81,33 | phenylcoumaran benzylic ether reductase | phenylcoumaran benzylic ether reductase-like protein | [65] |
| Contig_24773_bb |  |  | -17,71 | -3,03 | -36,04 | -4,79 |  | pyrophosphate-dependent phosphofructokinase beta subunit | phosphofructokinase V11 |  |
| Contig_50586_bb | 1,03 | 4,95 | 17,42 |  | 8,27 | 1,72 | 40,92 | formate dehydrogenase | phosphoglycerate dehydrogenase | [66,67] |
| Contig_36386_bb |  | -2,92 | -2,14 | -1,47 | -2,78 | -1,94 | -24,24 | protein |  |  |
| Contig_42492_bb | -1,89 | -1,31 | 1,21 | -1,19 | -1,12 | -1,50 | 31,97 | glucose-6-phosphate dehydrogenase |  |  |
| Contig_40856_bb | -1,31 | -1,24 | 1,09 | 1,03 | -1,12 | -1,14 | -31,09 | phospholipase d alpha | phospholipase D alpha | [7,68] |
| Contig_47044_bb | 8,60 | 1,58 |  | 20,33 | 2,34 | 13,64 |  | mip pip subfamily | PIP2 protein | [29] |
| Contig_17544_bb |  |  | -3,75 | -20,57 | -4,25 | -12,01 |  | plasma membrane h+ atpase | plasma membrane H+-ATPase | [29] |
| Contig_24980_bb |  | - | -6,98 | 1,26 | -21,97 | -1,32 |  | h(\+)-transporting atpase plant fungi plasma membrane |  |  |
| Contig_26543_bb |  |  | -6,07 | - | -5,98 | -20,90 |  | glycoside hydrolase family 28 protein | polygalacturonase | [3] |
| Contig_22317_bb |  | - |  | 29,82 | - | 19,68 |  | polygalacturonase-inhibiting protein | polygalacturonase-inhibiting protein | [69] |
| Contig_41987_bb | 1,76 | -1,80 |  | 35,24 | 1,05 | 5,71 |  | leucine rich repeat protein |  |  |
| Contig_1674_bb | -1,48 | -1,92 | 2,26 | 2,94 | -1,03 | 1,46 | 22,38 | profilin | profilin | [8,70,71] |
| Contig_2408_bb | -1,62 | -2,32 | 1,11 | 1,59 | -1,52 | 1,02 | 20,54 | protein | proline-rich protein |  |
| Contig_43920_bb | 3,15 | -1,39 |  | 24,39 | 1,20 | 7,76 |  | predicted protein [Populus trichocarpa] |  |  |
| Contig_13682_bb | -1,16 | -1,88 |  | 27,11 | 1,25 | 3,01 |  | root phototropism | putative callose synthase catalytic subunit | [1] |
| Contig_38613_bb | 1,11 | -1,05 |  | 21,68 | 2,01 | 2,91 |  | 60s ribosomal protein l7a |  |  |
| Contig_55680_bb | -34,36 |  | 2,96 | -4,41 | 1,63 | -11,67 |  | ccch-type zinc finger protein | putative CCCH-type zinc finger transcription factor |  |
| Contig_51206_bb | -6,61 |  | 20,46 |  | 5,32 | -2,70 |  | zinc finger c-x8-c-x5-c-x3-h type family protein |  |  |
| Contig_48641_bb | 11,13 | 3,05 |  |  | 4,79 | 22,38 |  | gtp-binding protein | putative GTP-binding protein | [29,72] |
| Contig_13040_bb |  |  | -1,45 | -1,40 | -1,59 | -1,54 | -20,34 | gtp binding protein |  |  |
| Contig_31658_bb | -1,48 | -1,31 | 26,04 | 23,04 | 1,96 | 2,01 |  | protein | putative leucine-rich repeat transmembrane protein |  |
| Contig_40422_bb | 1,43 | 13,71 |  |  | 28,50 | 2,41 |  | protein | putative membrane protein |  |
| Contig_37339_bb |  |  | -14,49 | -19,84 | -24,61 | -31,62 |  | protein | putative PDF1-interacting protein 1 |  |
| Contig_39340_bb |  | -3,94 |  | 20,33 | -2,01 | 5,93 |  | protein |  |  |
| Contig_11886_bb |  | - |  | 2,72 | -26,37 | 1,61 |  | potassium efflux antiporter | putative potassium transport protein | [2] |
| Contig_37713_bb | -1,06 | 1,18 | 1,33 | 1,07 | 1,23 | 1,04 | 20,46 | serine carboxypeptidase iii precursor | putative serine carboxypeptidase precursor | [15] |
| Contig_35609_bb | - | 1,21 |  | 16,26 | 1,66 | 27,73 |  | brassinosteroid-regulated protein bru1 | putative xyloglucan endotransglucosylase/hydrolase | [73,74] |
| Contig_49827_bb |  |  | -9,64 | -26,45 | -13,55 | -30,57 |  | pyrophosphate-energized vacuolar membrane proton | pyrophosphate-energized vacuolar membrane proton pump |  |
| Contig_32427_bb | 1,13 | -1,73 | -4,54 | -2,31 | -3,23 | -2,26 | 23,87 | vacuolar h+-translocating inorganic pyrophosphatase |  |  |
| step1_rep_c47766 |  |  | -2,68 | - | -3,29 | -20,34 |  | inorganic pyrophosphatase |  |  |
| Contig_12245_bb |  |  | -15,57 | - | -25,49 | - |  | at1g19870 f6f9_18 | QD31 (IQ-domain 31); calmodulin binding [Arabidopsis] |  |
| Contig_1677_bb |  |  | -8,23 | -45,18 | -13,73 | -73,99 |  | protein | receptor kinase | [75] |
| Contig_4080_bb |  | - |  | 25,75 | - | 17,00 |  | protein kinase chloroplast |  |  |
| Contig_46129_bb | 2,68 | 1,38 |  | 21,68 | 2,34 | 5,71 | - | 60s ribosomal protein l23 | ribosomal protein L14 |  |
| step1_rep_c47769 | 2,04 | -2,03 |  | 5,79 | -1,32 | 3,55 | 37,08 | s-adenosyl-l-homocysteine hydrolase | S-adenosyl-L-homocystein hydrolase |  |
| Contig_33613_bb | -55,79 | -7,21 | 1,03 | -7,55 | -1,70 | -15,18 |  | pectinesterase like protein | secretory laccase |  |
| Contig_26783_bb | 3,24 | -1,28 | 5,61 | 23,15 | 1,30 | 7,65 |  | pollen-specific protein |  |  |
| Contig_50659_bb | 2,48 | 1,19 | 14,02 | 29,28 | 2,07 | 5,74 | - | protein |  |  |
| Contig_24007_bb | -21,03 |  | -1,06 | -5,07 | -1,83 | -9,02 |  | leucine-rich repeat receptor-like protein kinase | ser-thr protein kinase | [75] |
| Contig_2569_bb | -9,90 |  | -5,65 | -42,61 | -7,98 | -35,87 |  | protein |  |  |
| Contig_43011_bb |  |  | - | -19,10 | -23,74 | -14,68 |  | receptor protein kinase perk1 | somatic embryogenesis receptor-like kinase 1 protein |  |
| Contig_51797_bb |  |  | -2,68 | -14,70 | -4,61 | -27,10 |  | protein kinase |  |  |
| Contig_20183_bb |  |  | -9,64 | -26,45 | -8,13 | -14,31 |  | protein kinase family protein |  |  |
| Contig_461_bb | -3,11 | 2,09 | 35,35 |  | 6,84 | -1,50 |  | cytochrome p450 | steroid 22-alpha-hydroxylase |  |
| step1_rep_c47643 |  |  | -101,44 | - | -166,11 | - |  | subtilase family protein | subtilisin-like protease |  |
| Contig_19300_bb | -17,10 |  | -1,30 | - | -1,93 | -36,71 |  | sucrose synthase 2 | sucrose synthase | [12,45,76,77,78,79] |
| Contig_56175_bb | - |  | -1,35 | -48,12 | -2,25 | -103,36 |  | sucrose synthase 2 |  |  |
| Contig_55525_bb | -39,78 |  | -1,46 | - | -2,29 | -92,62 |  | sucrose transporter 1 | sucrose transporter 1 | [2] |
| Contig_54626_bb |  |  | -15,57 | - | -25,49 | - |  | thaumatin-like protein | thaumatin-like protein TLP1 | [31] |
| Contig_44794_bb |  |  | -11,97 | -49,22 | -19,92 | -79,06 |  | ---NA--- | transcription factor DRE-binding factor 2 |  |
| Contig_48304_bb |  | -2,10 |  | 3,63 | -1,38 | 2,31 | 37,08 | protein | ubiquitin-conjugating enzyme E2 |  |
| Contig_46654_bb | 1,36 | -1,01 | 1,29 | 1,78 | 1,18 | 1,57 | -28,93 | dihydroflavonol reductase | UDP-D-apiose/UPD-D-xylose synthetase |  |
| Contig_11276_bb | 1,73 | 1,19 |  | 24,39 | 2,21 | 4,08 |  | protein | UNE1-like protein |  |
| Contig_26940_bb | -1,08 | -2,01 |  | 31,17 | 1,14 | 3,37 | 2,40 | vacuolar invertase | vacuolar invertase 1 | [8,21,80,81] |
| Contig_39711_bb | -131,99 |  | 1,66 | -6,02 | -1,09 | -15,89 | 1,48 | xyloglucan endotransglucosylase hydrolase protein 2 | xyloglucan endotransglucosylase/hydrolase | [3,12,29,73] |
| Contig_49319_bb | -1,99 | 2,07 | 39,07 |  | 5,68 | 1,05 |  | protein | unknown [Gossypium hirsutum] |  |
| Several contigs |  |  |  |  |  |  |  | UDP-glucose pyrophosphorylase |  | [82] |
| step1_rep_c48790 |  |  |  |  |  | 15.21 |  | Germin-like protein | GhGLP1, GhGLP2 | [83] |
| Contig_24637_bb | 1,32 | -1,04 |  | 2,55 | 1,34 | 1,88 | 25,57 | histone h1 | NO HIT Gossypium |  |
| Contig_55568_bb | -8,65 | -7,09 | 44,64 | 36,60 | 1,18 | 1,14 |  | hydrophobic protein lti6a | NO HIT gossypium |  |
| Contig_23889_bb | 1,67 | -1,93 |  | 29,82 | -1,01 | 5,47 |  | methyladenine glycosylase family protein | No HIT Gossypium |  |
| Contig_44063_bb | 1,84 | 4,88 | 50,42 | 19,05 | 8,84 | 3,28 | 1,21 | ---NA--- | NO HIT Gossypium |  |
| Contig_23718_bb |  | - |  | 46,08 | - | 30,41 |  | ---NA--- | NO HIT gossypium |  |
| Contig_3530_bb | -1,56 | 3,81 | 24,18 |  | 7,98 | 1,14 |  | non-phototropic hypocotyl 3 | NO HIT gossypium |  |
| step1_rep_c47781 | 7,23 | -1,11 |  | - | 1,45 | 21,46 |  | protease inhibitor seed storage lipid transfer protein family protein | No HIT Gossypium |  |
| Contig_42211_bb | 3,89 | 5,33 | -3,04 | -4,16 | 1,37 | 1,06 | -22,68 | protein | NO HIT Gossypium |  |
| Contig_53988_bb | 22,25 | 2,77 |  | 4,98 | 3,33 | 11,31 |  | protein | NO HIT gossypium |  |
| Contig_19047_bb | -14,40 |  | -1,91 | -41,87 | -2,83 | -27,49 |  | protein | NO HIT Gossypium |  |
| Contig_31857_bb | 1,55 | -2,53 |  | 36,60 | -1,30 | 6,06 |  | protein | NO HIT gossypium |  |
| Contig_34181_bb |  |  | -1,38 | -13,23 | -2,39 | -28,24 |  | protein | No HIT Gossypium |  |
| Contig_35142_bb |  |  | -2,83 | -27,18 | -4,77 | -49,70 |  | protein | No HIT Gossypium |  |
| Contig_6716_bb | 1,55 | -1,41 |  | 20,33 | 1,35 | 4,29 |  | af412084_1at3g11590 f24k9_26 | HIT Gossypium low cov% |  |
| Contig_43067_bb | -1,52 | -1,72 | 1,42 | 1,60 | -1,15 | 1,04 | 47,95 | aldehyde dehydrogenase | HIT Gossypium low cov% |  |
| step1_rep_c47680 | 1,11 | -1,64 |  | 20,33 | 1,28 | 3,43 | 17,90 | aldo keto | HIT Gossypium low cov% |  |
| Contig_37542_bb |  | -31,62 |  | 32,53 | -21,97 | 22,38 |  | asparagine synthetase | HIT Gossypium low cov% |  |
| Contig_24467_bb | -4,01 | -22,47 | -1,07 | 5,24 | -5,88 | 2,41 | 1,09 | extensin-like protein | HIT Gossypium low cov% |  |
| Contig_48886_bb |  | - |  | 6,80 | -26,37 | 3,70 | -3,13 | aspartyl protease | HIT Gossypium low cov% |  |
| Contig_54342_bb |  | - |  | 43,38 | -9,67 | 7,09 | 2,07 | aspartyl protease | HIT Gossypium low cov% |  |
| Contig_36160_bb | -1,97 | 1,20 | 22,33 |  | 3,27 | 1,23 |  | at1g72970 f3n23_17 | HIT Gossypium low cov% |  |
| Contig_34781_bb | -1,20 | 9,14 | -2,34 | -25,72 | -1,58 | -3,73 |  | at4g08810 t32a17_120 | HIT Gossypium low cov% |  |
| Contig_16608_bb | -28,93 |  | -1,61 | -17,63 | -2,59 | -24,10 |  | atsk41 (shaggy-like protein kinase 41) atp binding protein kinase protein serine threonine kinase | HIT Gossypium low cov% |  |
| Contig_35236_bb |  |  | -2,68 | - | -3,29 | -20,34 |  | ca2+ antiporter cation exchanger | HIT Gossypium low cov% |  |
| Contig_3269_bb |  |  | -13,42 | - | -21,97 | - |  | cobra-like 4 protein | HIT Gossypium low cov% |  |
| step1_rep_c47666 | -7,20 |  | -2,48 | -27,18 | -3,34 | -16,89 |  | cop1-interacting protein 7 | HIT Gossypium low cov% |  |
| Contig_36761_bb | 3,01 | 1,61 |  | 24,39 | 2,69 | 6,21 |  | diacylglycerol acyltransferase | HIT Gossypium low cov% |  |
| Contig_54407_bb | 1,05 | -1,04 |  | 5,11 | 1,74 | 2,15 | 33,24 | elongation factor 1- | HIT Gossypium low cov% |  |
| Contig_40023_bb |  |  | -12,31 | -8,45 | -21,97 | -13,56 |  | fructose- -bisphosphatase | HIT Gossypium low cov% |  |
| Contig_53371_bb |  | -39,52 | -10,74 | 2,04 | -21,97 | 1,31 |  | glutamate decarboxylase | HIT Gossypium low cov% |  |
| Contig_25280_bb | -11,25 |  | -4,20 | -143,99 | -5,97 | -49,73 |  | glycosyltransferase-like protein | HIT Gossypium low cov% |  |
| Contig_32592_bb | 1,06 | -2,44 | 10,28 | 26,56 | -1,13 | 4,10 | 1,43 | hth fad binding aldehyde-lyase mandelonitrile lyase | HIT Gossypium low cov% |  |
| Contig_45798_bb | - |  | 1,09 | -10,04 | -1,61 | -24,47 |  | kinase interacting family protein | HIT Gossypium low cov% |  |
| Contig_7037_bb | -5,40 |  | -2,00 | -20,57 | -2,55 | -11,43 |  | kinesin light | HIT Gossypium low cov% |  |
| Contig_21557_bb | 1,34 | -1,03 | 1,39 | 1,92 | 1,20 | 1,63 | -65,30 | late embryogenesis abundant protein lea14- | HIT Gossypium low cov% |  |
| Contig_11840_bb | 3,02 | 1,49 | 11,20 | 22,66 | 2,49 | 6,25 |  | mate efflux family protein | HIT Gossypium low cov% |  |
| Contig_17368_bb | 3,79 | 1,27 | 10,28 | 30,64 | 2,08 | 8,19 |  | mate efflux family protein | HIT Gossypium low cov% |  |
| step1_rep_c48046 | 1,02 | 4,44 | 35,35 |  | 8,77 | 1,82 | -44,58 | meiosis 5 | HIT Gossypium low cov% |  |
| step1_rep_c47633 | 55,08 | 6,29 |  | 5,44 | 7,59 | 24,61 | -26,59 | ---NA--- | HIT Gossypium low cov% |  |
| step1_rep_c47720 | 31,16 | - |  |  | - | 50,11 | - | ---NA--- | HIT Gossypium low cov% |  |
| step1_rep_c47700 | -1,35 | 1,35 | 22,33 |  | 3,19 | 1,71 |  | ---NA--- | HIT Gossypium low cov% |  |
| Contig_51464_bb | -21,70 |  | 2,80 |  | -1,08 | -2,51 |  | nac domain ipr003441 | HIT Gossypium low cov% |  |
| Contig_56300_bb |  | -2,13 |  | 4,43 | -1,36 | 2,66 | -20,34 | nadh cytochrome b5 reductase | HIT Gossypium low cov% |  |
| Contig_27729_bb |  |  | -9,44 | -103,59 | -10,40 | -33,53 |  | o-acetyltransferase family protein | HIT Gossypium low cov% |  |
| Contig_34783_bb |  |  | -6,43 | -26,45 | -5,42 | -11,02 |  | o-acetyltransferase family protein | HIT Gossypium low cov% |  |
| Contig_17645_bb | - |  | -1,65 | -29,38 | -2,77 | -59,86 |  | oxygen-evolving enhancer protein chloroplast | HIT Gossypium low cov% |  |
| Contig_26653_bb |  |  | -9,12 | -12,49 | -15,83 | -20,34 |  | pectinacetylesterase family protein | HIT Gossypium low cov% |  |
| Contig_36974_bb |  |  | -11,27 | - | -9,23 | -24,86 |  | phosphoinositide phosphatase family protein | HIT Gossypium low cov% |  |
| Contig_38532_bb | 1,39 | 11,43 |  |  | 23,94 | 2,37 |  | proliferation-associated protein 2g4 | HIT Gossypium low cov% |  |
| Contig_52657_bb | -32,54 | -65,87 | -2,62 | -1,29 | -6,38 | -2,34 | - | protein | HIT Gossypium low cov% |  |
| Contig_17935_bb | 2,13 | 3,48 | -2,36 | -3,85 | 1,05 | -1,30 | -26,59 | protein | HIT Gossypium low cov% |  |
| Contig_9806_bb | 1,44 | 1,59 | 4,07 | 3,68 | 2,40 | 2,33 | 37,08 | protein | HIT Gossypium low cov% |  |
| Contig_42317_bb | 1,11 | -1,12 | 1,80 | 2,24 | 1,25 | 1,61 | 33,88 | protein | HIT Gossypium low cov% |  |
| step1_rep_c47622 | 1,51 | 12,66 | - |  | 25,86 | 2,55 | -6,17 | protein | HIT Gossypium low cov% |  |
| step1_rep_c47830 | -1,80 | - | 20,46 |  | 25,08 | -1,23 | - | protein | HIT Gossypium low cov% |  |
| Contig_49168_bb |  | - |  | 21,68 | -14,95 | 7,10 |  | protein | HIT Gossypium low cov% |  |
| Contig_259_bb | 2,96 | - |  |  | 21,66 | 3,55 |  | protein | HIT Gossypium low cov% |  |
| Contig_174_bb |  |  | - | -13,23 | - | -20,34 |  | protein | HIT Gossypium low cov% |  |
| Contig_1806_bb | 1,26 | -1,79 | 10,28 | 23,15 | 1,14 | 4,03 |  | protein | HIT Gossypium low cov% |  |
| Contig_23380_bb | - | 1,22 |  |  | 1,66 | 23,27 |  | protein | HIT Gossypium low cov% |  |
| Contig_23720_bb | - |  | -2,22 | -14,20 | -3,83 | -27,11 |  | protein | HIT Gossypium low cov% |  |
| Contig_28820_bb | -41,59 |  | -1,12 | - | -1,76 | -80,20 |  | protein | HIT Gossypium low cov% |  |
| Contig_35943_bb | -16,20 |  | -1,96 | - | -2,90 | -47,44 |  | protein | HIT Gossypium low cov% |  |
| Contig_36934_bb | 11,93 | -1,34 |  | 59,64 | 1,14 | 25,79 |  | protein | HIT Gossypium low cov% |  |
| Contig_41014_bb |  |  | -13,03 | - | -21,39 | - |  | protein | HIT Gossypium low cov% |  |
| step1_rep_c47904 | -1,98 |  | 20,46 |  | 2,65 | 1,33 |  | protein | HIT Gossypium low cov% |  |
| step1_rep_c48092 | 11,68 | 5,33 |  |  | 8,36 | 21,46 |  | protein | HIT Gossypium low cov% |  |
| Contig_42821_bb | - |  | -2,11 | -55,10 | -3,52 | -106,18 |  | protein | HIT Gossypium low cov% |  |
| Contig_20237_bb |  |  | -16,10 | -5,51 | -29,88 | -8,76 |  | secondary cell wall-related glycosyltransferase family 8 | HIT Gossypium low cov% |  |
| Contig_35613_bb | -1,30 | -1,97 | 24,18 | 36,60 | 1,26 | 2,85 | -3,13 | transferase family protein | HIT Gossypium low cov% |  |
| step1_rep_c47732 | -1,75 | 25,14 | - |  | 74,08 | -1,06 | -21,90 | unknown [Populus trichocarpa] | HIT Gossypium low cov% |  |
| Contig_30688_bb | 15,02 | 1,29 |  | - | 1,99 | 38,46 |  | nadh ubiquinone oxidoreductase-related | HIT Gossypium low cov% |  |
| Contig_35654_bb | 1,19 | -2,01 | -4,21 | -1,76 | -3,12 | -1,84 | 36,44 | vitamin-b12 independent methionine 5-methyltetrahydropteroyltriglutamate-homocysteine | HIT Gossypium low cov% |  |
| Contig_19479_bb | - |  | -2,44 | -60,24 | -4,05 | -112,96 |  | secondary cell wall-related glycosyltransferase family 8 | No HIT Gossypium low cov% secondary cell wall-related glycosyltransferase family 8 [Populus] |  |
| Contig_14773_bb | 11,13 | 1,17 |  | - | 1,84 | 29,52 |  | protein | NO HIT gossypium, acyltransferase, putative [Ricinus communis] |  |

* Not shown step1_rep_c47607 annotated as FbLate-2 protein (1363 reads exclusively from 22 dpa (fold-ratio not calculated), including 335 for Gh22 and 1028 for Gb22)

**References** (numbers may differ from numbers in the main text)

1. Ruan YL, Xu SM, White R, Furbank RT (2004) Genotypic and developmental evidence for the role of plasmodesmatal regulation in cotton fiber elongation mediated by callose turnover. Plant Physiology 136: 4104-4113.

2. Ruan YL, Llewellyn D, Furbank RT (2001) The control of single-celled cotton fiber elongation by developmentally reversible gating of plasmodesmata and coordinated expression of sucrose and K^+^ transporters and expansin. The Plant cell 13: 47-60.

3. Al-Ghazi Y, Boutrot S, Arioli T, Dennis ES, Llewellyn D (2009) Transcript profiling during fiber development identifies pathways in secondary metabolism and cell wall structure that may contribute to cotton fiber quality. Plant and Cell Physiology 50: 1364-1381.

4. Shimizu Y, Aotsuka S, Hasegawa O, Kawada T, Sakuno T, et al. (1997) Changes in levels of mRNAs or cell-call-related enzymes in growing cotton fibers. Plant and Cell Physiology 38: 375-378.

5. Chen X, Guo W, Liu B, Zhang Y, Song X, et al. (2012) Molecular mechanisms of fiber differential development between G. barbadense and G. hirsutum revealed by genetical genomics. PLoS ONE 7: e30056.

6. Shi H, Wang X, Li D, Tang W, Wang H, et al. (2007) Molecular characterization of cotton 14-3-3L gene preferentially expressed during fiber elongation. J Genet Genomics 34: 151-159.

7. Yang YW, Bian SM, Yao Y, Liu JY (2008) Comparative proteomic analysis provides new insights into the fiber elongating process in cotton. J Proteome Res 7: 4623-4637.

8. Zhao PM, Wang LL, Han LB, Wang J, Yao Y, et al. (2010) Proteomic identification of differentially expressed proteins in the Ligon lintless mutant of upland cotton (*Gossypium hirsutum* L.). J Proteome Res 9: 1076-1087.

9. Sun G, Xie F, Zhang B (2011) Transcriptome-wide identification and stress properties of the 14-3-3 gene family in cotton (Gossypium hirsutum L.). Funct Integr Genomics 11: 627-636.

10. Qin YM, Ma Pujol F, Shi YH, Feng JX, Liu YM, et al. (2005) Cloning and functional characterization of two cDNAs encoding NADPH-dependent 3-ketoacyl-CoA transferase fromd developing cotton fibers. Cell Research 15: 465-473.

11. Qin YM, Hu CY, Pang Y, Kastaniotis AJ, Hiltunen JK, et al. (2007) Saturated very-long-chain fatty acids promote cotton fiber and Arabidopsis cell elongation by activating ethylene biosynthesis. Plant Cell 19: 3692-3704.

12. Alabady M, Youn E, Wilkins TA (2008) Double feature selection and cluster analyses in mining of microarray data from cotton. BMC Genomics 9: 295.

13. Zhu YQ, Xu KX, Luo B, Wang JW, Chen XY (2003) An ATP-binding cassette transporter GhWBC1 from elongating cotton fibers. Plant Physiology 133: 580-588.

14. Grula JW, Hudspeth RL, Hobbs SL, Anderson DM (1995) Organization, inheritance and expression of acetohydroxyacid synthase genes in the cotton allotetraploid Gossypium hirsutum. Plant Molecular Biology 28: 837-846.

15. Li C-H, Zhu Y-Q, Meng Y-L, Wang J-W, Xu K-X, et al. (2002) Isolation of genes preferentially expressed in cotton fibers by cDNA filter arrays and RT-PCR. Plant Science 163: 1113-1120.

16. Li XB, Fan XP, Wang XL, Cai L, Yang WC (2005) The cotton ACTIN1 gene is functionally expressed in fibers and participates in fiber elongation. Plant Cell 17: 859-875.

17. Chi J, Wang X, Zhou H, Zhang G, Sun Y, et al. (2008) Molecular cloning and characterization of the actin-depolymerizing factor gene in Gossypium barbadense. Genes Genet Syst 83: 383-391.

18. Wang HY, Wang J, Gao P, Jiao GL, Zhao PM, et al. (2009) Down-regulation of GhADF1 gene expression affects cotton fibre properties. Plant Biotechnol J 7: 13-23.

19. Hou L, Luo XY, Wang WF, Xiao YH, Luo M, et al. (2007) Cloning, expression and characterization of an ADP-ribosylation factor gene from cotton (*Gossypium hirsutum* L.). Acta Agronominca Sinica 08.

20. Harmer SE, Orford SJ, Timmis J (2002) Characterization of six alpha-expansin genes in *Gossypium hirsutum* (upland cotton). Molecular Genetics and Genomics: 09-janv.

21. Gou JY, Wang LJ, Chen SP, Hu WL, Chen XY (2007) Gene expression and metabolite profiles of cotton fiber during cell elongation and secondary cell wall synthesis. Cell Research 17: 422-434.

22. Feng JX, Ji SJ, Shi YH, Xu Y, Wei G, et al. (2004) Analysis of five differentially expressed gene families in fast elongating cotton fiber. Acta Biochim Biophys Sin (Shanghai) 36: 51-56.

23. Orford SJ, Timmis JN (1998) Specific expression of an expansin gene during elongation of cotton fibres. Biochim Biophys Acta 1398: 342-346.

24. Whittaker DJ, Triplett BA (1999) Gene-specific changes in alpha-tubulin transcript accumulation in developing cotton fibers. Plant Physiology 121: 181-188.

25. Li XB, Cai L, Cheng NH, Liu JW (2002) Molecular characterization of the cotton *GhTUB1* gene that is preferentially expressed in fiber. Plant Physiology 130: 666-674.

26. Park W, Scheffler BE, Bauer PJ, Campbell BT (2010) Identification of the family of aquaporin genes and their expression in upland cotton (Gossypium hirsutum L.). BMC Plant Biology 10: 142.

27. Tang G, Zhu X, Gakiere B, Levanony H, Kahana A, et al. (2002) The bifunctional LKR/SDH locus of plants also encodes a highly active monofunctional lysine-ketoglutarate reductase using a polyadenylation signal located within an intron. Plant Physiology 130: 147-154.

28. Turley RB, Taliercio E (2008) Cotton benzoquinone reductase: up-regulation during early fiber development and heterologous expression and characterization in Pichia pastoris. Plant Physiol Biochem 46: 780-785.

29. Arpat AB, Waugh M, Sullivan JP, Gonzales M, Frisch D, et al. (2004) Functional genomics of cell elongation in developing cotton fibers. Plant Molecular Biology: 911-929.

30. Hovav R, Udall JA, Hovav E, Rapp R, Flagel LE, et al. (2008) A majority of cotton genes are expressed in single-celled fiber. Planta 227: 319-329.

31. Tu LL, Zhang XL, Liang SG, Liu DQ, Zhu LF, et al. (2007) Genes expression analyses of sea-island cotton (*Gossypium barbadense* L.) during fiber development. Plant Cell Reports 26: 1309-1320.

32. Kim HJ, Triplett BA, Zhang HB, Lee MK, Hinchliffe DJ, et al. (2012) Cloning and characterization of homeologous cellulose synthase catalytic subunit 2 genes from allotetraploid cotton (Gossypium hirsutum L.). Gene 494: 181-189.

33. Iqbal S, Bashir A, Naseer HM, Ahmed M, Malik KA (2008) Identification of differentially genes in developing cotton fibers (*Gossypium hirsutum* L.) through differential display. Journal of Biotechnology: 1-10.

34. Zhang D, Hrmova M, Wan CH, Wu C, Balzen J, et al. (2004) Members of a new group of chitinase-like genes are expressed preferentially in cotton cells with secondary walls. Plant Molecular Biology 54: 353-372.

35. Betancur L, Singh B, Rapp RA, Wendel JF, Marks MD, et al. (2010) Phylogenetically distinct cellulose synthase genes support secondary wall thickening in arabidopsis shoot trichomes and cotton fiber. Journal of Integrative Plant Biology 52: 205-220.

36. Yuan D, Tu L, Zhang X (2011) Generation, annotation and analysis of first large-scale expressed sequence tags from developing fiber of *Gossypium barbadense* L. PLoS ONE 6: e22758.

37. Mei W, Qin Y, Song W, Li J, Zhu Y (2009) Cotton GhPOX1 encoding plant class III peroxidase may be responsible for the high level of reactive oxygen species production that is related to cotton fiber elongation. J Genet Genomics 36: 141-150.

38. Nicol F, Hofte H (1998) Plant cell expansion: scaling the wall. Current Opinion in Plant Biology 1: 12-17.

39. Ruan XM, Luo F, Li DD, Zhang J, Liu ZH, et al. (2011) Cotton BCP genes encoding putative blue copper-binding proteins are functionally expressed in fiber development and involved in response to high-salinity and heavy metal stresses. Physiol Plant 141: 71-83.

40. Shen F, Yu S, Han X, Fan S (2004) Cloning and characterization of a gene encoding cysteine proteases from senescent leaves of *Gossypium hirsutum*. Chinese Science Bulletin 49: 2601-2607.

41. Li HB, Qin YM, Pang Y, Song WQ, Mei WQ, et al. (2007) A cotton ascorbate peroxidase is involved in hydrogen peroxide homeostasis during fibre cell development. New Phytol 175: 462-471.

42. Ferguson DL, Turley RB, Kloth RH (1997) Identification of a delta-TIP cDNA clone and determination of related A and D genome subfamilies in Gossypium species. Plant Molecular Biology 34: 111-118.

43. John ME, Crow LJ (1992) Gene expression in cotton (*Gossypium hirsutum* L.) fiber: cloning of the mRNAs. Proc Natl Acad Sci USA 89: 5679-5773.

44. John ME (1996) Structural characterization of genes corresponding to cotton fiber mRNA, E6: reduced E6 protein in transgenic plants with antisense gene. Plant Molecular Biology 30: 297-306.

45. Haigler C, Zhang D, Wilkerson CG (2005) Biotechnological improvement of cotton fiber maturity. Physiologia plantarum 124: 285-294.

46. Huang GQ, Xu WL, Gong SY, Li B, Wang XL, et al. (2008) Characterization of 19 novel cotton FLA genes and their expression profiling in fiber development and in response to phytohormones and salt stress. Physiol Plant 134: 348-359.

47. Liu D, Tu L, Li Y, Wang L, Zhu L, et al. (2008) Genes encoding fascilin-like Arabinogalactan proteins are specifically expressed during cotton fiber development. Plant Molecular Biology Reporter 26: 98-113.

48. Indrais E, Cheema HMN, Samas A, Bashir A (2011) Temporal expression analysis and cloning of cotton (*Gossypium hirsutum*) fiber genes. International Journal of Agriculture and Biology 13: 89-94.

49. Rinehart JA, Petersen MW, John ME (1996) Tissue-specific and developmental regulation of cotton gene FbL2A. Demonstration of promoter activity in transgenic plants. Plant Physiology 112: 1331-1341.

50. Wu Z, Soliman KM, Bolton JJ, Saha S, Jenkins JN (2008) Identification of differentially expressed genes associated with cotton fiber development in a chromosomal substitution line (CS-B22sh). Funct Integr Genomics 8: 165-174.

51. Shi Y-H, Zhu SW, Mao XZ, Feng JX, Qin YM, et al. (2006) Transcriptome profiling, molecular biological, and physiological studies reveal a major role for ethylene in cotton fiber cell elongation. The Plant cell: 651-664.

52. Yajun H, Wangzhen G, Xinlian S, Tianzhen Z (2008) Molecular cloning and characterization of a cytosolic glutamine synthetase gene, a fiber strength-associated gene in cotton. Planta 228: 473-483.

53. John ME, Keller G (1995) Characterization of mRNA for a proline-rich protein of cotton fiber. Plant Physiology 108: 669-676.

54. Ji S-J, Lu Y, Feng J-X, Wei G, Li J, et al. (2003) Isolation and analyses of genes prefentially expressed during early cotton fiber development by subtractive PCR and cDNA array. Nucleic Acids Research 31: 2534-2543.

55. Gao P, Zhao PM, Wang J, Wang HY, Wu XM, et al. (2007) Identification of genes preferentially expressed in cotton fibers: A possible role of calcium signaliing in cotton fiber elongation. Plant Science: 61-69.

56. Preuss ML, Delmer DP, Liu B (2003) The cotton kinesin-like calmodulin-binding protein associates with cortical microtubules in cotton fibers. Plant Physiol 132: 154-160.

57. Ma DP, Tan H, Si Y, Creech RG, Jenkins JN (1995) Differential expression of a lipid transfer protein gene in cotton fiber. Biochimica et Biophysica Acta 1257.

58. Hudspeth RL, Hobbs SL, Anderson DM, Rajasekaran K, Grula JW (1996) Characterization and expression of metallothionein-like genes in cotton. Plant Molecular Biology 31: 701-705.

59. Suo J, Liang X, Pu L, Zhang Y, Xue Y (2003) Identification of GhMYB109 encoding a R2R3 MYB transcription factor that expressed specifically in fiber initials and elongating fibers of cotton (*Gossypium hirsutum* L.). Biochim Biophys Acta 1630: 25-34.

60. Loguercio LL, Zhang JQ, Wilkins TA (1999) Differentiated regulation of six novel MYB-domain genes defines two distinct expression patterns in allotetraploid cotton (*Gossypium hirsutum* L.). Molecular and General Genetics 261: 660-671.

61. Cedroni ML, Cronn RC, Adams KL, Wilkins TA, Wendel JF (2003) Evolution and expression of MYB genes in diploid and polyploid cotton. Plant Mol Biol 51: 313.

62. Meng C, Cai C, Zhang T, Guo W (2009) Characterization of six novel NAC genes and their responses to abiotic stresses in Gossypium hirsutum L. Plant Science 176: 352-359.

63. Wang H, Guo Y, Lv F, Zhu H, Wu S, et al. (2010) The essential role of GhPEL gene, encoding a pectate lyase, in cell wall loosening by depolymerization of the de-esterified pectin during fiber elongation in cotton. Plant Molecular Biology 72: 397-406.

64. Yang L, Zhu H, Guo W, Zhang T (2010) Molecular cloning and characterization of five genes encoding pentatricopeptide repeat proteins from Upland cotton (Gossypium hirsutum L.). Mol Biol Rep 37: 801-808.

65. Turley R (2008) Expression of a phenylcoumaran benzylic ether reductase-like protein in the ovules of *Gossypium hirsutum*. Biologia Plantarum 52: 759-762.

66. Chaudhary B, Hovav R, Rapp R, Verma N, Udall JA, et al. (2008) Global analysis expression in cotton fibers from wiild and domesticated *Gossypium barbadense*. Evolution and Development 10: 567-582.

67. Wang QQ, Liu F, Chen XS, Ma XJ, Zeng HQ, et al. (2010) Transcriptome profiling of early developing cotton fiber by deep-sequencing reveals significantly differential expression of genes in a fuzzless/lintless mutant. Genomics 96: 369-376.

68. Wanjie SW, Welti R, Moreau RA, Chapman KD (2005) Identification and quantification of glycerolipids in cotton fibers: reconciliation with metabolic pathway predictions from DNA databases. Lipids 40: 773-785.

69. Shi H, Zhu L, Zhou Y, Li G, Chen L, et al. (2009) A cotton gene encoding a polygalacturonase inhibitor-like protein is specifically expressed in petals. Acta Biochim Biophys Sin (Shanghai) 41: 316-324.

70. Wang J, Wang HY, Zhao PM, Han LB, Jiao GL, et al. (2010) Overexpression of a profilin (GhPFN2) promotes the progression of developmental phases in cotton fibers. Plant and Cell Physiology 51: 1276-1290.

71. Argiriou A, Kalivas A, Michailidis G, Tsaftaris A (2012) Characterization of PROFILIN genes from allotetraploid (Gossypium hirsutum) cotton and its diploid progenitors and expression analysis in cotton genotypes differing in fiber characteristics. Mol Biol Rep 39: 3523-3532.

72. Delmer D, Pear J, Andrawis A, Stalker D (1995) Genes encoding small GTP-binding proteins analogous to mammalian rac are preferentially expressed in developing cotton fibers. Molecular and General Genetics MGG 248: 43-51.

73. Lee J, Burns TH, Light G, Sun Y, Fokar M, et al. (2010) Xyloglucan endotransglycosylase/hydrolase genes in cotton and their role in fiber elongation. Planta 232: 1191-1205.

74. Shao MY, Wang XD, Ni M, Bibi N, Yuan SN, et al. (2012) Regulation of cotton fiber elongation by xyloglucan endotransglycosylase/hydrolase genes. Genet Mol Res 10.

75. Li YL, Sun J, Xia GX (2005) Cloning and characterization of a gene for an LRR receptor-like protein kinase associated with cotton fiber development. Molecular Genetics and Genomics 273: 217-224.

76. Zhu H, Han X, Lv J, Zhao L, Xu X, et al. (2011) Structure, expression differentiation and evolution of duplicated fiber developmental genes in Gossypium barbadense and G. hirsutum. BMC Plant Biology 11: 40.

77. Ruan Y-L, Llewellyn D, Furbank RT (2003) Suppression of sucrose synthase gene expression represses cotton fiber initiation, elongation and seed development. The Plant cell: 952-964.

78. Brill E, van Thournout M, White RG, Llewellyn D, Campbell PM, et al. (2011) A novel isoform of sucrose synthase is targeted to the cell well during secondary cell wall synthesis in cotton fiber. Plant Physiology 157: 40-54.

79. Jiang Y, Guo W, Zhu H, Ruan YL, Zhang T (2011) Overexpression of GhSusA1 increases plant biomass and improves cotton fiber yield and quality. Plant Biotechnol J.

80. Wang L, Li XR, Lian H, Ni DA, He YK, et al. (2010) Evidence that high activity of vacuolar invertase is required for cotton fiber and Arabidopsis root elongation through osmotic dependent and independent pathways, respectively. Plant Physiology 154: 744-756.

81. Sergeeva LI, Keurentjes JJ, Bentsink L, Vonk J, van der Plas LH, et al. (2006) Vacuolar invertase regulates elongation of *Arabidopsis thaliana* roots as revealed by QTL and mutant analysis. Proc Natl Acad Sci USA 103: 2994-2999.

82. Wang QH, Zhang X, Li FG, Hou YX, Liu XL, et al. (2011) Identification of a UDP-glucose pyrophosphorylase from cotton (*Gossypium hirsutum* L.) involved in cellulose biosynthesis in *Arabidopsis thaliana*. Plant Cell Reports 30: 1303-1312.

83. Kim H, Triplett BA (2004) Cotton fiber germin-like protein. I. Molecular cloning and gene expression. Planta 218: 516-524.
