# Supplementary material for: Deep Sequencing Reveals Differences in the Transcriptional Landscapes of Fibers from Two Cultivated Species of Cotton
Source: PLoS One. 2012 Nov 15;7(11):e48855. doi: 10.1371/journal.pone.0048855 (PMC3499527; doi:10.1371/journal.pone.0048855)
Supplement: Table S8 — Mapping results of EST-SSR and EST-SNP markers. Chromosome localization of segregating loci are indicated (“unl” indicates an unlinked locus in JoinMap software). (DOC) [file pone.0048855.s010.doc]

**Table S8: Mapping results of EST-SSR and EST-SNP markers**. Chromosome localization of segregating loci are indicated, with “unl” indicating an unlinked locus in JoinMap software.

| Marker | Contig | locus | Chr. | Annotation |
| --- | --- | --- | --- | --- |
| CIR420 | Contig_10581_bb | CIR420a | c8 | plasma membrane intrinsic protein |
| CIR420 |  | CIR420b | c24 |  |
| CIR426 | Contig_21747_bb | CIR426a | c3 | leaf senescence |
| CIR426 |  | CIR426b | c14 |  |
| CIR429 | Contig_24435_bb | CIR429 | c21 | protein |
| CIR430 | Contig_24773_bb | CIR430 | c19 | pyrophosphate-dependent phosphofructokinase beta subunit |
| CIR431 | Contig_25250_bb | CIR431 | c13 | cbs domain-containing protein |
| CIR432 | Contig_2636_bb | CIR432 | c21 | binding protein |
| CIR434^§^ | Contig_26800_bb | CIR434a | c14 | at1g68530 t26j14_10 |
| CIR434 |  | CIR434b | c26 |  |
| CIR438 | Contig_29034_bb | CIR438 | c20 | gtp-binding protein |
| CIR443 | Contig_31710_bb | CIR443a | c6 | protein |
| CIR443 |  | CIR443b | c6 |  |
| CIR444 | Contig_31711_bb | CIR444 | c23 | pectinesterase family protein |
| CIR452 | Contig_34451_bb | CIR452 | c8 | aldo keto |
| CIR455 | Contig_35943_bb | CIR455 | c25 | protein |
| CIR458 | Contig_37372_bb | CIR458 | unl | protein |
| CIR463 | Contig_39536_bb | CIR463 | c13 | homeobox-leucine zipper protein hat7 |
| CIR466 | Contig_40907_bb | CIR466 | unl | hypothetical protein |
| CIR467 | Contig_41402_bb | CIR467a | c5 | 5 start site is |
| CIR467 |  | CIR467b | c19 |  |
| CIR469 | Contig_41751_bb | CIR469a | unl | protein |
| CIR469 |  | CIR469b | c20 |  |
| CIR471 | Contig_41903_bb | CIR471 | c11 | protein |
| CIR477 | Contig_44955_bb | CIR477 | c24 | atp-binding cassette subfamily member abc component protein 10 |
| CIR481 | Contig_46349_bb | CIR481a | c5 | stromal cell-derived factor 2 precursor |
| CIR481 |  | CIR481b | c16 |  |
| CIR483 | Contig_48376_bb | CIR483a | c8 | unknown [Populus trichocarpa] |
| CIR483 |  | CIR483b | c24 |  |
| CIR485 | Contig_49503_bb | CIR485 | c12 | protein |
| CIR487 | Contig_50023_bb | CIR487 | c10 | beta ig-h3 domain-containing protein |
| CIR489 | Contig_52479_bb | CIR489a | c4 | glutathione s-transferase |
| CIR489 |  | CIR489b | c22 |  |
| CIR492 | Contig_53851_bb | CIR492 | c25 | protein |
| CIR494 | Contig_55128_bb | CIR494a | c17 | endo-beta -glucanase |
| CIR494 |  | CIR494b | c2 |  |
| CIR496 | Contig_6462_bb | CIR496 | unl | alpha l-fucosidase 2 |
| CIR503 | step1_rep_c47923 | CIR503a | c6 | serine-threonine kinase |
| CIR503 |  | CIR503b | unl |  |
| CIR505 | step1_rep_c48906 | CIR505 | c8 | glycosyl hydrolase family 17 protein |
| SNP1 | Contig_23072_bb | SNP1 | c11 | gras family transcription factor |
| SNP2^§^ | Contig_26800_bb | SNP2 | c14 | at1g68530 t26j14_10 |
| SNP3 | Contig_26848_bb | SNP3 | c6 | heat shock protein 70 |
| SNP4 | Contig_27026_bb | SNP4 | c15 | elongation factor 1- |
| SNP5 | Contig_27860_bb | SNP5 | c17 | cinnamyl-alcohol dehydrogenase |
| SNP6 | Contig_32067_bb | SNP6 | (c6)* | membrane protein |
| SNP7 | Contig_33456_bb | SNP7 | c20 | cytosolic phosphoglucomutase |
| SNP8 | Contig_33511_bb | SNP8a | c22 | pfkb-type carbohydrate kinase family protein |
| SNP8 |  | SNP8c | c4 |  |
| SNP11 | Contig_37983_bb | SNP11 | c3 | syp61 (syntaxin of plants 61) snap receptor |
| SNP12 | Contig_38860_bb | SNP12 | c14 | integral membrane family protein |
| SNP15 | Contig_42665_bb | SNP15 | c1 | proton pump interactor |
| SNP16 | Contig_42803_bb | SNP16 | c3 | protein |
| SNP17 | Contig_43877_bb | SNP17 | c2 | alpha-glucan-protein synthase |
| SNP19 | Contig_47754_bb | SNP19a | c20 | integral membrane |
| SNP19 |  | SNP19b | c10 |  |
| SNP20 | Contig_48233_bb | SNP20 | c15 | endo-1,3-1,4-beta-d-glucanase |
| SNP22 | Contig_49921_bb | SNP22 | c19 | stearoyl-acyl-carrier protein desaturase |
| SNP24 | Contig_51213_bb | SNP24a | unl | protein |
| SNP24 |  | SNP24b | unl |  |
| SNP25 | Contig_5257_bb | SNP25 | c11 | salicylic acid-induced fragment 1 protein |
| SNP26 | Contig_53096_bb | SNP26 | c4 | nuclear acid binding |
| SNP27 | Contig_53370_bb | SNP27 | c11 | zinc finger |
| SNP28 | Contig_53515_bb | SNP28 | c25 | cathepsin b |
| SNP32 | Contig_9663_bb | SNP32 | c9 | quasimodo1-like protein |
| SNP34 | Contig_23539_bb | SNP34 | c13 | pleckstrin homology domain-containing protein |
| SNP36 | Contig_17742_bb | SNP36 | c19 | white-brown-complex abc transporter family |
| SNP37 | Contig_19300_bb^£^ | SNP37 | (c6)* | sucrose synthase |
| SNP39 | Contig_23308_bb | SNP39 | c19 | acid phosphatase class b family protein |
| SNP40 | Contig_26940_bb | SNP40 | c14 | vacuolar invertase |
| SNP41 | Contig_28608_bb | SNP41 | c8 | tubulin alpha 3 |
| SNP44 | Contig_55680_bb | SNP44 | c9 | ccch-type zinc finger protein |
| SNP45 | Contig_56175_bb^£^ | SNP45 | c25 | sucrose synthase |
| SNP47 | step1_rep_c48885 | SNP47 | c19 | alpha expansin |

* assignation to c6 putative, ^£^ 2 contigs with similar annotation (sucrose synthase) and high similarity (96%) that had best hit with isoform SusC [Brill et al. (2011)], ^§^ Two markers, CIR434 and SNP2, were designed from the same contig (Contig_26800_bb) and, expectedly, mapped a locus at the same position on chromosome 14.
